# Effects of Competition on Left Prefrontal and Temporal Cortex During Conceptual Comparison of Brand-Name Product Pictures: Analysis of fNIRS Using Tensor Decomposition †

**DOI:** 10.3390/brainsci15020127

**Published:** 2025-01-28

**Authors:** Terrence M. Barnhardt, Jasmine Y. Chan, Behnaz Ghoraani, Teresa Wilcox

**Affiliations:** 1 Department of Psychology, Florida Atlantic University, Boca Raton, FL 33431, USA; tbarnhardt@fau.edu (T.M.B.); chanj2018@fau.edu (J.Y.C.); 2 Department of Electrical Engineering and Computer Science, Florida Atlantic University, Boca Raton, FL 33431, USA; bghoraani@fau.edu

**Keywords:** semantic memory, semantic cognition, inferior frontal gyrus, anterior temporal lobe, functional near-infrared spectroscopy, fNIRS, tensor decomposition, situated cognition, brand-name products, consumer neuroscience

## Abstract

Background/Objectives: Recent theories of the neurocognitive architecture of semantic memory have included a distinction between semantic control in the left inferior frontal gyrus (LIFG) and semantic representation in the left anterior temporal lobe (LATL). Support for this distinction has been found both in tasks in which high semantic selection demands have been instantiated and in tasks in which previous presentations of semantic information that compete with target information have been instantiated. Methods: In the current study, these manipulations were combined in a novel manner into a single task in which brand-name product pictures were used. Functional near-infrared spectroscopy (fNIRS) was used to measure hemodynamic activity and tensor decomposition, in addition to grand averaging, was used to analyze the fNIRS output. Results: Both analytic methods converged on the same set of findings. That is, in line with past studies, greater activity in the LIFG was observed in the competitive condition than in a repeated condition. However, unlike past studies, greater activity in the competitive condition was also observed in both the left and right anterior temporal lobes (ATLs). Conclusions: While it was possible that the novel combination of high selection and competition into a single task unlocked a semantic selection mechanism in the bilateral ATL, a number of other post-hoc explanations were offered for this unusual finding, including a re-interpretation of the high-selection task as an ad hoc categorization task. Finally, the convergence of the tensor decomposition and grand averaging approaches on the same set of findings supported tensor decomposition as a viable approach to the analysis of fNIRS data.

## 1. Introduction

Semantic memory is our permanent store of knowledge regarding concepts, categories, objects, facts, and words [1]. Knowledge of a concept (e.g., croissant) consists not only of its features (e.g., taste, texture, shape, smell, composition, etc.), but also of its associative relationships with other concepts (e.g., croissants go with butter and jam) and its conceptual similarity with other concepts (e.g., bread, buns, scones, etc.; [2]). In contrast, semantic cognition refers to our abilities to both store and retrieve concepts in support of behavioral and language comprehension and production [3]. A key issue in the study of semantic cognition is understanding its capacity for the contextually determined flexible retrieval of concepts from semantic memory. For example, *car* can be associated with traffic jams and slow driving, or *car* can be associated with racing and fast driving, depending upon whether the context is being on holiday or being at the race track [4]. Using one of the examples detailed below, Elmer’s Glue can be associated with Imperial Sugar when thinking about things that are white or Elmer’s Glue can be associated with Mazola corn oil when thinking about things that are liquid. The overall purpose of the present study was to probe the semantic memory system—especially the left inferior frontal gyrus and left anterior temporal lobe—in order to determine the roles these structures played in a task that demanded flexible retrieval from semantic memory. In the present study, pictures of brand-name products served as the stimuli (instead of words in printed text), fNIRS was used to measure hemodynamic activity (instead of functional magnetic resonance imaging [fMRI]), and tensor decomposition was used to analyze the fNIRS output (in addition to grand averaging).

### 1.1. Cognitive Neuropsychology Investigations of Semantic Cognition

Over the last four decades, there has been intense interest in delineating the neural structures that support semantic memory and semantic cognition [5]. Initially, neuropsychological studies of semantic dementia supported the claim that semantic memory was localized in the left temporal lobe [6]. Damage to the left anterior temporal lobe (LATL) results in taxonomic errors (e.g., “horse”, “animal”) when naming a target picture (e.g., CAMEL) and the concepts to which an individual semantic dementia patient makes these types of errors are consistent across a variety of tasks, reflecting a semantic storage deficit [4]. However, evidence began to accumulate that the semantic memory system was also instantiated in additional brain regions besides the left temporal lobe. For example, neuropsychological studies of semantic aphasia supported the claim that semantic memory retrieval was localized in the left inferior frontal gyrus (LIFG; [7]). Damage to the left LIFG results in thematic errors (e.g., “zebra”, “pyramids”) when naming a target picture (e.g., CAMEL), and the concepts to which an individual semantic aphasia patient makes these types of errors are inconsistent across tasks and are disproportionally reduced by facilitative phonological cues, reflecting a semantic access deficit [8]. This distinction between semantic representation and semantic control is a central feature of what Lambon-Ralph et al. [3] have termed the controlled semantic cognition framework. With this framework in mind, the more specific purpose of the present study was to investigate, in a task that demanded flexible retrieval from semantic memory, whether the LIFG is indeed the locus of a semantic cognition retrieval mechanism and the LATL is the locus of the semantic memory store.

### 1.2. Cognitive Neuroscience Investigations of Selection Demands During Semantic Retrieval

Like research with semantic aphasia patient populations, positron emission tomography and fMRI studies with unimpaired populations have also found that the LIFG is activated in tasks involving semantic memory retrieval [9,10]. However, Thompson-Schill et al. [11] argue that semantic retrieval tasks often also include the need to select a particular aspect of semantic knowledge from amongst competing, alternative aspects in order to make a task-appropriate response. For example, in verb generation tasks, participants must select just one response (e.g., “tie”) out of many possible responses (e.g., tie, hang, swing, climb, jump, etc.) to the noun *rope*. Thus, increasing semantic retrieval and LIFG activation by increasing the amount of semantic information that is retrieved in response to a cue can often be reinterpreted in terms of increasing selection demands. In order to disentangle these alternative conceptualizations, a number of strategies have been employed to both instate and manipulate the degree of selection demands in tasks involving semantic retrieval.

#### Manipulating Selection Demands During Semantic Retrieval

One approach to operationalizing selection demands is to alter a global similarity task, such as the Pyramids and Palm Trees Test [12], the Camel and Cactus Test [13], or the low-selection semantic comparison condition used by Thompson-Schill et al. [11]. In the low-selection semantic comparison condition, participants selected which one of two comparison concepts (e.g., tick or school) was more similar to a cue concept (e.g., flea; all stimuli presented in printed text). In this task, there are so many dimensions (e.g., size, animacy, animal group, etc.) and attributes (e.g., small, live, insect, etc.) that are appropriately relevant that there is little need to select any one dimension or attribute in order to correctly respond with “tick”. In order to instate a greater degree of selection demands in the low-selection semantic comparison task, Thompson-Schill et al. [11] developed a high-selection semantic comparison (HS-SC) condition, in which participants again selected which one of two comparison concepts (e.g., tongue or bone) was more similar to a cue concept (e.g., tooth), but in this condition they had to do so after being prompted with a particular dimension (e.g., Color). Note that, in order to properly complete the HS-SC task, the participant must not only isolate one dimension (e.g., Color) from amongst many possible dimensions on which the cue item could be matched with one of the alternative choices (e.g., Body Part, Function, Composition, Size, etc.) but must also select one value (i.e., one attribute; e.g., white) from amongst many possible values on that dimension. That is, the participant must focus on the fact that the color of a tooth is white, the color of a bone is white, and the color of a tongue is pink in order to select “bone” as the correct response. Indeed, the HS-SC condition was associated with greater activation than the low-selection condition in the LIFG, but not in the LATL. To investigate the distinction between the semantic memory store in the LATL and the semantic cognition retrieval mechanism in the LIFG, the present experiment used the HS-SC task to instate selection demands shown to be associated with increased activity in the LIFG.

Another strategy for instating selection demands in a semantic retrieval task has been to increase competition for the selection of semantic information that is currently relevant to a target by emphasizing, in a previous presentation, the selection of other semantic information also relevant to that same target. For example, in the Different condition of a word generation task, Thompson-Schill et al. [14] first had participants generate an Action response to the noun *dollar* (e.g., “spend”) and then later generate a Color response to the noun *dollar* (e.g., “green”). Even though the selection of an appropriate Color response would, in isolation, hypothetically involve selection demands, Thompson-Schill et al. [14] reasoned that these selection demands would be magnified in the Different condition because of the presence of the previously activated, but no longer relevant, semantic information. Indeed, they observed increased activation in the LIFG in the Different condition relative to a non-repeated word generation condition. They also observed decreased activation in both the LIFG and temporal lobe in the Same condition, in which the same dimension (e.g., Action) was prompted in both exposures to the target concept. Most interestingly for the present purposes, they also observed decreased activation in the left temporal lobe in the Different condition. This pattern of results provided strong support for the distinction between the localization of the semantic representation of the target concept in the temporal lobe in both the Same and Different conditions (i.e., the target concept was repeated in both the Same and Different conditions and produced deactivation in both conditions) and the localization of the selection of semantic information relevant to that concept in the LIFG (i.e., the selection of relevant semantic information was repeated in the Same condition, resulting in deactivation, but switched in the Different condition, resulting in increased activation).

### 1.3. The Current Experiment

As noted earlier, the present experiment used the HS-SC task in order to instate selection demands shown to be associated with increased activity in the LIFG. In addition, to further increase the selection demands, a Different (i.e., switch) condition was included in the HS-SC task. The idea was that, in the Different condition, previously presented semantic information relevant to the target would compete with semantic information that was currently relevant to the target. Using the example of an HS-SC trial that was presented earlier, a participant would be first prompted with the cue dimension Color, after which, they would need to select which one of two comparison concepts (e.g., tongue or bone) was more similar to the target cue concept (e.g., tooth) on that dimension (correct answer: bone). Then, after a lag that included several other HS-SC trials, the participant would be presented with the same stimulus triplet a second time, but be prompted with the cue dimension Body Part, thereby switching the semantic information relevant to the target cue concept (i.e., tooth) from Color to Body Part and the subsequent correct response from “bone” to “tongue”. Behavioral performance and hemodynamic activity in the Different condition were compared to performance in two other conditions: a Same condition in which the same triplet was presented with the same cue dimension twice and a Once condition in which a triplet was presented with a cue dimension a single time. Harking back to the introductory paragraph, notice that the Different condition instated the type of flexible semantic retrieval demands that should provide some insight into exactly how the semantic cognition system supports this key ability.

#### Prior Switch Results and Predictions for the Current Experiment

To our knowledge, this is the first experiment in which the switch manipulation has been used in combination with the HS-SC task in order to instate a high degree of selection demands. Many other switch manipulations have been employed [15,16,17], and in all of these, greater activation in the switch condition than in the repetition and control conditions was observed in the LIFG. None of these experiments reported greater activation in the switch condition in the right inferior frontal gyrus (RIFG), LATL, or right anterior temporal lobe (RATL).

One of the manipulations most similar to the current manipulation was employed by Badre et al. [18]. In their Congruent condition, they employed a HS-SC task (e.g., which one of two comparison concepts—coal or leek—is more similar to a cue concept—tar—after being prompted with the cue dimension Color). In addition, in their Incongruent condition, they increased selection demands even further by using lures that were highly associated with the cue concepts (e.g., which one of two comparison concepts—jade or league—is more similar to a cue concept—ivy—after being prompted with the cue dimension Color). Notice in the Incongruent condition, that if the cue dimension had not been presented and the participant was making their choice on the basis of global similarity, their choice would be “league” rather than “jade”. Thus, the use of “league” as a lure in the Incongruent condition instated an even higher degree of competition with the correct response “jade” than was found in the Congruent condition. They found greater activation in the LIFG to the Incongruent condition than in the Congruent condition. No activation difference between these conditions was found in the RIFG or temporal lobe.

To summarize, greater LIFG activation has been observed (1) in a HS-SC condition compared to a low-selection semantic comparison condition [11], (2) in switch conditions compared to repetition and control conditions across a variety of tasks and domains [15,16,17,19], and (3) in an Incongruent HS-SC condition compared to a Congruent HS-SC condition [18]. In none of these experiments was a similar difference observed in the RIFG or left or right anterior temporal lobes (ATLs). Given these findings, it was expected that employing a HS-SC task with a switch manipulation (i.e., the Different condition) would produce greater LIFG activation than repeated (i.e., Same) or control (i.e., Once) conditions, while producing equivalent activation across those conditions in other areas of the cortex, such as the RIFG, LATL, and RATL.

### 1.4. Other Novel Aspects of the Current Experiment

#### 1.4.1. Pictures of Brand-Name Products

In addition to this being the first experiment to use a switch manipulation in conjunction with the HS-SC task, there are several other novel aspects of this experiment that should be noted. First, many of the prior experiments exploring the neurocognitive basis of semantic cognition have used words presented as printed text. In contrast, in the present experiment, pictures of brand-name products were used. Brand-name products are probably the most common objects/concepts with which we come into contact every day. Brand-name product categories show typicality effects, such as those found in common, nominal concepts [20]. Thus, it seems safe to assume that knowledge of brand-name products, such as knowledge of common, nominal concepts, is stored in semantic memory. As such, these type of stimuli provides an opportunity to determine whether conclusions regarding semantic cognition that are drawn from research with words presented as printed text extend to research in which concepts with proper names (i.e., brand-name products) are presented in a picture format.

#### 1.4.2. Rationale for fNIRS Imaging

Second, in general, much of the imaging work investigating semantic cognition has used fMRI, as has most of the work in consumer neuroscience [21]. In contrast, the present research used an optical imaging technique known as fNIRS to measure localized changes in relative levels of oxygenated (HbO) and deoxygenated (HbR) hemoglobin concentrations in cortical regions of the brain. Even though fNIRS is able to measure blood flow only in targeted areas of the cerebral cortex, fNIRS has the potential to provide a more cost-effect, more tolerable, and more generalizable method of measuring hemodynamic response than fMRI, more generalizable because it can be used in more naturalistic settings (e.g., while the participant is mobile; [22]).

#### 1.4.3. Rationale for the Use of Tensor Decomposition Instead of Grand Averaging

Finally, instead of using grand averaging to extract the main patterns in the hemodynamic signal obtained with fNIRS, the present research used an alternative and relatively novel technique known as tensor decomposition (TD) [23]. In both grand averaging and TD, the fNIRS signal is averaged across multiple blocks in the same experimental condition in order to compute a hemodynamic response function (HRF) for each condition in each channel in each participant. Then, in grand averaging, the HbO values at the time points from the HRF that fall within a time of interest (TOI) are averaged to produce a single mean HbO value for each condition/channel/participant. Finally, the mean values of all conditions/channels that are in a region of interest (ROI) are averaged to produce a single mean value for each participant in each condition in each ROI. It is these values that are then subjected to statistical analysis.

There are several well-known weaknesses in the grand averaging approach. First, defining the TOIs and ROIs is often informed not only by visual inspection of the HRF, but also by patterns of data observed in prior experiments, and by theoretical expectations about what the HRF should look like and where in the brain it should be observed. Second, there is a tremendous loss of information about the temporal shape of the HRF and the spatial distribution of the HRF when the data are collapsed across both time points and channels before it is subjected to statistical analysis.

In contrast, TD examines the simultaneous interaction of the entire time signature of the HRF (without selecting a TOI and reducing it to a single mean value), the distribution of the HRF across all the channels (without selecting an ROI and collapsing it across the individual channels in the ROI to obtain a single mean value), and experimental manipulations to statistically determine the time, channel, and condition combinations (i.e., components) that make up the totality of the hemodynamic signal. In TD, patterns (i.e., components) combining time, space, and condition emerge from the data, rather than being determined by visual inspection and prior research, both of which can be influenced by subjective considerations.

Although TD has been applied to fNIRS with infants [24,25], it has not, to our knowledge, been applied to fNIRS in adults. In addition, there are a number of differences between the approach used in the infant paper and the approach used here. Given that the TD approach to fNIRS data analysis is so new, it seemed wise to compare the TD results to grand averaging results in order to validate the outcome of the TD and to understand its relation to the overall hemodynamic signal. For this reason, there will be a small section in the Results that will compare the results of the TD to grand averaging.

## 2. Materials and Methods

### 2.1. Participants

The behavioral data and fNIRS measurements were analyzed from a total of 48 participants (31 female; age in years *M* = 20.06, *SD* = 5.48; range = 18 to 50) from Texas A&M University’s and Florida Atlantic University’s undergraduate participant pools. The experiment was approved by the two universities’ Institutional Review Boards. Data were collected from a total of 51 participants, but 3 were excluded due to incomplete data collection. An a priori power analysis was performed using Cohen’s *d* to estimate sample size for a paired samples design with two groups. Based on pilot studies in our lab, a small to moderate effect size (0.35), a power of 0.75, and a directional *p* of 0.05 were used. The analysis indicated a sample size of 44 was required. The sample size of 48 was used so that one participant filled each cell of the completely counterbalanced design. All the participants had either normal or corrected-to-normal vision, gave informed consent, and were given course credit or extra credit for their 1.5 h-long experiment participation.

### 2.2. Experimental Design and Procedures

The experiment was programmed with E-Prime 3.0 software [26]. Stimuli were presented on a Dell computer that had an Intel Core i7 with 8.00 GB RAM and a 64-bit operating system that was running Windows 10. The entire experiment consisted of two phases, a study phase and a test phase. In the study phase, the HS-SC task was presented and, in the test phase, a two-alternative, forced-choice purchase intention task was presented. Only the study phase method and results are presented here. During the instructions for the study phase, participants were told that the purpose of the HS-SC task was to investigate their perception of common, everyday objects. No mention was made that the objects were brand-name products or that there would be a second phase consisting of a purchase intention test.

The within-subjects independent variable was Type of Processing (i.e., Once vs. Same vs. Different). In the Once condition, participants processed triplets of brand-name products once in the HS-SC task. In the Same condition, participants processed triplets of brand-name products twice in the HS-SC task, with the same cue dimension used both times for any single triplet. In the Different condition, participants processed triplets of brand-name products twice in the HS-SC task, but with a different cue dimension used during the second presentation of the same triplet. Regardless of the Type of Processing condition, different cue dimensions were used across different triplets (see Section 2.3).

**Figure 1:**
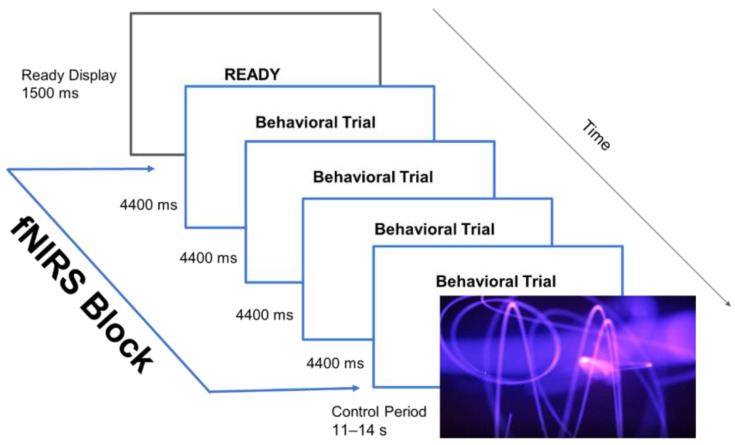
An fNIRS block, along with the ready display and the control period.

A block design was used during the study task. An fNIRS block consisted of four behavioral HS-SC trials (detailed below), lasting a total of ~18 s. The length of the fNIRS blocks was set such that there was enough time for the particular type of processing in that particular condition to begin and stabilize. FNIRS blocks alternated with control periods (see Figure 1). The control period was the presentation of a visually engaging, animated video clip that allowed for HbO in the semantic system to return to a resting state before the onset of the next fNIRS block. For example, a video clip called “purple electricity” showed purple streaks moving across the screen in various directions on a black background. There were three different video clips presented throughout the study task. The length of the video clips was varied from 12 s to 14 s long in order to reduce the likelihood of an anticipatory hemodynamic response for each of the fNIRS blocks. No single video clip was presented in two control periods in a row.

In order to instate the Type of Processing conditions in the study task, there were 20 fNIRS blocks: four Once, four first-time Same, four second-time Same (Same, for short), four first-time Different, and four second-time Different (Different, for short). There was one order of Type of Processing conditions across the fNIRS blocks in the study phase (see Table S1). For both the Same and Different conditions, the lag between repeated triplets varied from two fNIRS blocks to eight fNIRS blocks, or an average of five fNIRS blocks. Taking into account the ready display and control period associated with each fNIRS block, this meant that the average lag was approximately 2.5 min. No Type of Processing condition was presented twice in a row in the study task.

**Figure 2:**
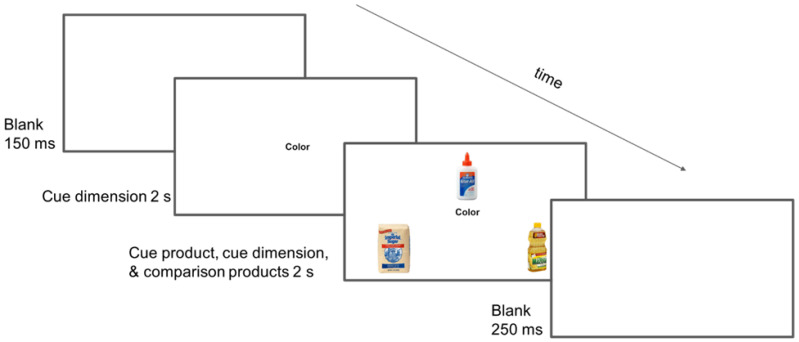
A high-selection semantic comparison (HS-SC) behavioral trial. A magnified version of the third display screen can be viewed in Supplemental materials (Figure S1).

See Figure 2 for an example of a HS-SC behavioral trial that was presented during the study phase. Which comparison product was presented on which side of the screen was counterbalanced across participants. The participants were instructed to select the comparison brand-name product that fit the combination of the cue dimension (e.g., Color) and the cue brand-name product (e.g., Elmer’s Glue). Participants pressed the “D” key for the left comparison brand-name product (e.g., Imperial Sugar was the correct choice) or the “L” key for the right comparison brand-name product. The participant’s response did not terminate the screen presenting the brand-name products. Instead, that display terminated at 2 s, regardless of whether the participant responded sooner or later, in order to maintain the 4.4 s for each behavioral trial and 17.6 s for each fNIRS block. Late responses were counted as errors.

The behavioral dependent variables for the study phase were accuracy and RT. Accuracy was defined as the proportion of trials on which a correct response was given. RT was defined as the amount of time it took, in ms, for a participant to make a button press after the onset of the presentation of the screen displaying the brand-name products.

At the hemodynamic level, in addition to the Type of Processing factor, hemisphere was also often included in order to compare Type of Processing effects across hemispheres in either frontal regions or temporal regions. The dependent variable at the hemodynamic level was relative changes in HbO as measured by fNIRS. In order to determine the hemodynamic response in a particular Type of Processing condition, the HbO response was averaged across the four fNIRS blocks in that condition.

### 2.3. Materials

Each HS-SC trial included one of two brand-name product pictures from the same product category that served as a cue product (e.g., Elmer’s Glue or RoseArt Glue) and two brand-name product pictures that served as comparison products (e.g., Imperial Sugar pure cane sugar and Mazola corn oil). In addition, each such group of stimuli included two cue dimensions for use when that group of stimuli was presented in the Different condition (e.g., Color [pictured in Figure 2] and State of Matter [not pictured in Figure 2]). To be clear, when such a group of stimuli was presented in the Different condition, the cue product remained the same across the two presentations, and only the cue dimension changed. The cue product within a particular pair that was presented (e.g., either Elmer’s Glue or RoseArt Glue) was counterbalanced across participants. For the sake of convenience, we refer to these six stimuli (i.e., two cue products, two comparison products, and two cue dimensions) as a stimulus sextuplet.

Given that there were 4 fNIRS blocks with 4 behavioral trials each in each of the 3 Type of Processing conditions, 48 sextuplets were needed for the study task. This constructed 2 groups of 48 sextuplets, yielding a total of 96 sextuplets. Which group of 48 was presented and which 16 sextuplets of the 48 were presented in which Type of Processing condition was counterbalanced across participants. No product category (e.g., glue, sugar, cooking oil) or brand-name (e.g., Elmer’s, RoseArt, Imperial Sugar, Mazola) was used more than once in the stimulus set.

The cue dimensions varied from trial to trial in the HS-SC task, even within the same fNIRS block. Overall, 45 different cue dimensions were used, with Purpose (*n* = 27), Size (*n* = 23), Setting (*n* = 23), Shape (*n* = 18), State of Matter (*n* = 15), and Body Part (*n* = 10) occurring most frequently amongst the 192 cue dimensions that were used. The brand-name product names and the associated cue dimensions for all 96 sextuplets are presented in Table S2.

### 2.4. FNIRS Instrumentation, Configuration, and Preprocessing

**Figure 3:**
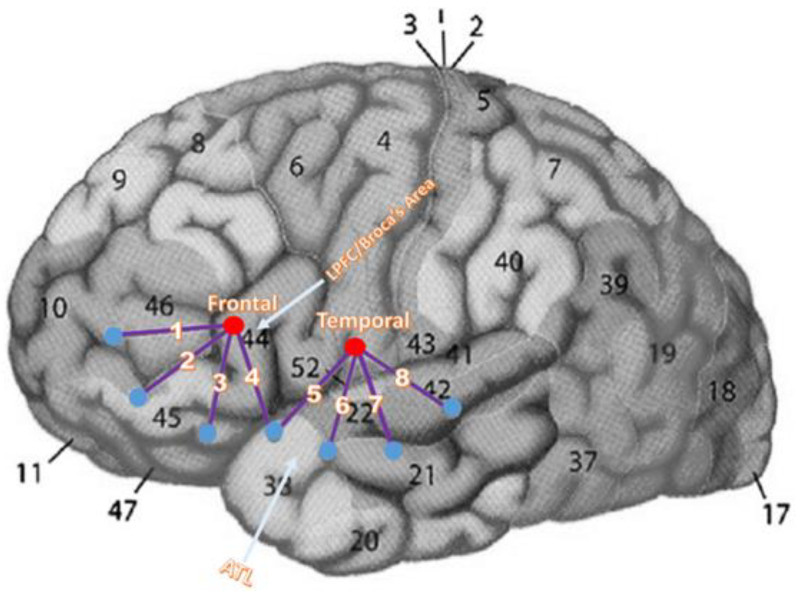
FNIRS probe design. Only the left hemisphere is shown (analogous areas were measured in the right hemisphere). Red dots: sources; blue dots: detectors; purple lines: channels; white numbers: channel numbers; black numbers: Brodmann’s areas.

FNIRS was used to measure changes in hemodynamic response. The fNIRS system consisted of a control box and fiber optic cables attached to the infrared light sources (i.e., emitters) and detectors. Probe geometry (see Figure 3) consisted of 4 sources and 14 detectors that were embedded into a hand-made headgear. A figure depicting the average optode location digitization associated with the placement of this headgear on experiment participants can be found in the Supplemental Materials (see Figure S2). The MNI coordinates of these average optode locations can also be found in the Supplemental Materials (see Table S3). The distance between a source and its associated detectors was 3 cm. Each source emitted near-infrared light in a continuous wave at 690 nm to measure HbR and at 830 nm to measure HbO (TechEN Inc., Milford, MA, USA, CW7 System). Each detector recorded near-infrared light in both wavelengths. The near-infrared light was square wave modulated at 4–12 kHz, such that each laser had a unique frequency to prevent crosstalk from multiple sources. The sampling rate was 25 Hz.

Preprocessing of neuroimaging data was conducted with Homer2 [27], an open software that was programmed with MATLAB R2016a [28]. The processing of raw data started by converting the intensity of the photons to optical density. The quality of each channel was checked and pruned from the analysis (dRange = 1 × 10^3^ to 1 × 10^7^, SNRthresh = 2, SDrange = 0.0 to 45.0, reset = 0). Motion artifacts were detected and corrected using principal component analysis (PCA; nSV = 0.80). Any additional motion artifacts that were not corrected with PCA were identified on a channel-by-channel basis and excluded from the analysis (tMotion = 0.5, tMask = 1.0, STDEVthresh = 20.0, AMPthresh = 3.5). Trials with motion artifacts occurring between 2 s before stimulus onset and 18 s after stimulus onset were excluded from the analysis. Additionally, the signal was bandpass filtered using a third-order Butterworth filter with a 0.01 Hz high-pass filter and a 0.5 Hz low-pass filter to process out low-frequency drifts and high-frequency noise (i.e., physiological interference). The optical density of the signal was converted to concentration with the modified Beer–Lambert law (ppf = 6.0 6.0). The HRF was created from 2 s before stimulus onset to 31 s after stimulus onset. Within each participant, the HbO values were averaged across the four fNIRS blocks in each Type of Processing condition.

### 2.5. Tensor Decomposition

The main goal of using TD was to identify the spatial and time signatures of the Type of Processing effect (i.e., the differences between the Same, Different, and Once conditions). A tensor is an array with three or more dimensions [29,30]. In order to represent the hemodynamic signal in the present experiment, a three-dimensional tensor was constructed by using three of the dimensions present in the hemodynamic signal: the distribution of the signal across time, the distribution of the signal across channels, and the distributions of the signal across subjects in each of the three Types of Processing conditions. TD then extracts the patterns underlying the multidimensional signal by compressing the information into components [23,31]. Any single component will be a unique combination of three subcomponents, which are typically referred to as modes (i.e., time, spatial, and subject) such that that component accounts for a percent of the variance in the overall signal, as represented by the tensor. (See Figure S3 in Supplemental Materials for a pictorial illustration of tensor construction and decomposition.) Some of the most important features particular to the manner in which TD was used in the present experiment are covered next. Additional technical details regarding the implementation of TD in the present experiment can be found in Appendix A.

The TD was programmed on MATLAB 2022b [32] using the Tensorlab 3.0 toolbox [33]. Missing data in the signal were substituted with the median values within the according condition and channel. Only fNIRS recordings from stimulus onset (0 s) to 20 s after stimulus onset were used so that information from the control period did not impact the components being extracted.

Given the exploratory nature of the current experiment, canonical polyadic decomposition (CPD) was used because less prior knowledge of the signal is needed compared to other forms of TD (e.g., Tucker decomposition; [23]). In the present experiment, the only assumption made was that there would be an effect of the experimental manipulation in the subject mode. No assumptions were made about the size or direction of the effect in the subject mode, the shape of the time mode, or the distribution across channels in the spatial mode.

It is not unusual for TD to produce a large number of components (e.g., 100 or more) in order to account for 100% of the variance in the hemodynamic signal, with some components accounting for a very small percent of the variance (e.g., 0.1%). In the present experiment, only components that accounted for at least 2% of the variance were considered in order to focus attention on components with relatively greater effect sizes. Typically, only the first seven components met this criterion. The rationale for focusing on components that accounted for at least 2% of the variance in the tensor was twofold. First, given that this was a “first of its kind” application of TD to adult fNIRS data, it seemed reasonable to focus on the “important” features of the hemodynamic signal as revealed by TD, and effect size is one common definition of importance. Second, we assumed that the composition of the grand-averaged hemodynamic signal would be more highly determined by relatively large TD components and that relatively small TD components would be masked by those larger components in the grand-averaged signal. Since one purpose of the present study was to validate the application of TD to adult fNIRS data by comparing the TD components to the grand-averaged signal, and only the large components would be transparently represented in the grand-averaged signal, it also made sense from this perspective to focus on the relatively large TD components.

TD will attempt to capture all possible interactions between the modes, producing relatively nonunique components, which can lead to model overfit. In order to counteract the possibility of model overfit, two strategies were employed. First, an orthogonality constraint was placed on the spatial mode in order to avoid nonunique components [33,34]. Second, multiple runs of the TD were performed, with a random initialization point for each run, so that unique components that were consistently emerging across runs could be identified. In order to identify the components that were consistently emerging, a k-means cluster analysis was performed on all of the components from all of the runs in order to group similar components into seven clusters. Then, the distance between the centroid and its components was calculated for each cluster. This distance was then summed for components in the same run to reveal the one run that was most representative of the clusters. The one run whose components had the shortest total distance from the centroids was selected to be analyzed.

Two tensors were constructed and decomposed using the study phase hemodynamic signal, one for the left hemisphere and one for the right hemisphere. Then, one run was selected from the left hemisphere decomposition and one from the right hemisphere decomposition, in accord with the procedure described above. As noted earlier, the main goal of using TD was to identify the spatial and time signatures of the Type of Processing effect (i.e., the differences between the Same, Different, and Once conditions). With this in mind, separate repeated measures ANOVAs were performed on the subject mode of each of the components derived from each of the two decompositions in order to select those components with a significant Type of Processing effect in the subject mode in each hemisphere. A critical analytical question was whether the selected components—independently derived for each hemisphere—were significantly different across the hemispheres. In the event that one of the hemispheres did not yield any components with a subject mode with a significant Type of Processing effect, the component from that hemisphere that had a time mode with a shape that closely matched the time mode of the significant component in the other hemisphere was chosen for comparison. A pipeline for the preprocessing, TD, and grand averaging of the fNIRS signal is displayed in Supplemental Materials Figure S4.

## 3. Results

### 3.1. Tensor Decomposition Results

**Figure 4:**
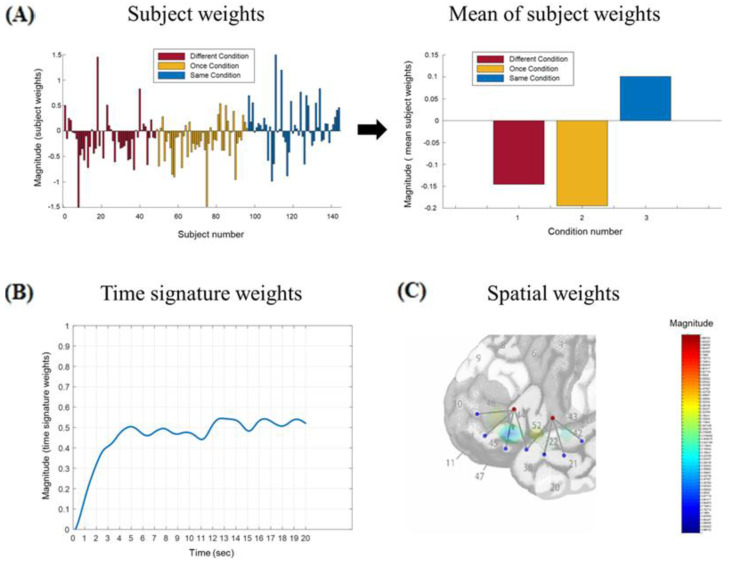
Left hemisphere component 1. (**A**) Subject mode weights and the mean of those weights for each condition. (**B**) Time mode weights. (**C**) Pictorial illustration of the spatial mode weights. In the spatial weights figure, the scale ranged from +1 at the top (depicted in dark red), to 0 in the middle (depicted in green), to −1 at the bottom (depicted in dark blue). A magnified version of subfigure (**C**) can be viewed in Supplemental materials (Figure S5).

The results from the cluster analysis revealed that the components in run 6 of the left hemisphere tensor and run 5 of the right hemisphere tensor best represented the components consistently emerging across different runs in the respective hemispheres. For left hemisphere run 6, each of the subject modes of the seven components was evaluated with a one-way repeated measures ANOVA in order to select any component(s) that had a significant effect of Type of Processing. Two components had a significant Type of Processing effect: the first component, *F*(2,94) = 8.63, *p* < 0.01, *MSE* = 0.14, *partial eta squared* = 0.16, and the third component, *F*(2,94) = 3.70, *p* = 0.03, *MSE* = 1.04, *partial eta squared* = 0.07. These two components accounted for 26% and 6% of the variance in the left hemisphere tensor, respectively. A pictorial representation of the first left hemisphere component can be seen in Figure 4. For right hemisphere TD run 5, there was a significant Type of Processing effect in the first component, *F*(2,94) = 3.97, *p* = 0.02, *MSE* = 0.91, *partial eta squared* = 0.08, and a marginally significant Type of Processing effect in the sixth component *F*(2,94) = 3.13, *p* = 0.05, *MSE* = 0.54, *partial eta squared* = 0.06. These two components accounted for 22% and 3% of the variance in the right hemisphere tensor, respectively.

**Figure 5:**
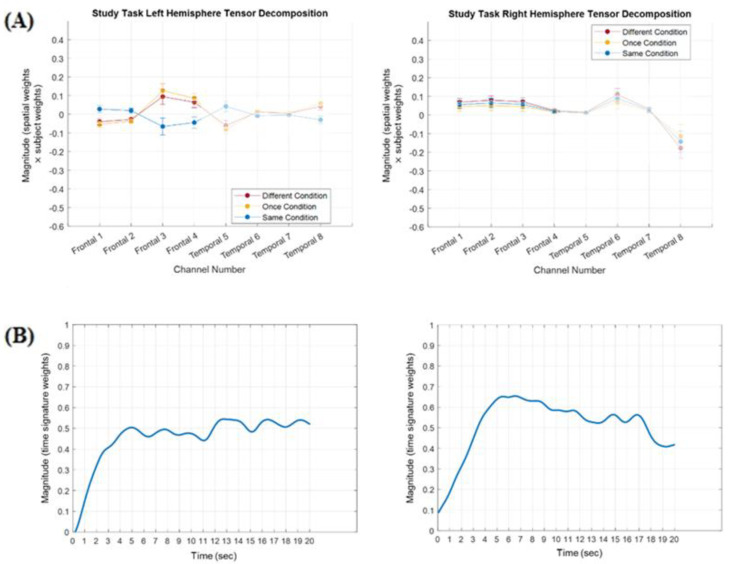
Left hemisphere frontal component 1 versus right hemisphere component 2. (Panel **A**) In each component, the subject weights were multiplied by the spatial (channel) weight to obtain the values displayed in each channel. In order to highlight that these were frontal components, the temporal channels have been faded. (Panel **B**) The time modes for the two components. *n* = 48. Error bars: ±1 SE.

The analytic strategy was to then compare the left and right frontal components with significant Type of Processing effects and the left and right temporal components with significant Type of Processing effects. However, neither the sign nor magnitude of the subject weights nor the spatial weights by themselves, reflected the sign or magnitude of the hemodynamic response. In order to accurately reflect the magnitude and direction of the hemodynamic response, the subject weights were multiplied by the spatial weights in each of the selected components. The manner in which this was accomplished is described in Appendix B. For example, in left hemisphere component one (see Figure 4), when the negative subject mode weights in the Different and Once conditions were multiplied by the negative spatial weights in channels 3 and 4, the result was positive subject x spatial weights in those conditions in those channels (see Figure 5). These subject x spatial weights profiles were then examined in order to determine whether the Type of Processing effects had been observed primarily in frontal channels, temporal channels, or both. The largest differences for left hemisphere component 1 were in frontal channels 3–4, for left hemisphere component 3 they were in temporal channels 5–8, and for both of the right hemisphere components they were in temporal channels 5–8. In addition, the differences in the subject x spatial weights in these channels afforded a more direct comparison to differences in the grand-averaged hemodynamic response in those channels in order to assess the validity of the TD approach.

#### 3.1.1. Frontal Cortex Components Analysis

One of the main hypotheses in the present experiment was that the role of the LIFG is to select one meaning from among the alternatives. Based on this, we predicted that activity in the Different condition would be greater than in the Same condition in the LIFG, but not in the RIFG. Given that there was no right hemisphere component with a significant subject mode in the frontal channels, the right hemisphere components were examined for a component with a time mode similar to that for left hemisphere component 1. There was only one: right hemisphere component 2. Left hemisphere component 1, right hemisphere component 2, and their respective time modes are displayed in Figure 5. A 3 (Type of Processing) × 2 (hemisphere) × 2 (channel 3 vs. channel 4) repeated measures ANOVA was conducted on the subject × spatial weights of these components. There was a significant Type of Processing × hemisphere interaction, *F*(2,94) = 9.31, *p* < 0.01, *MSE* = 0.04, *partial eta squared* = 0.17. Pairwise comparisons revealed that the Same condition (*M* = −0.06, *SE* = 0.04) was significantly less than the Different (*M* = 0.08, *SE* = 0.04, *d* = 0.51) and Once conditions (*M* = 0.11, *SE* = 0.03, *d* = 0.52), *p*’s < 0.01. At the same time, there were no differences across conditions in the right hemisphere; all *p*’s > 0.38. This result was consistent with a priori predictions regarding hemodynamic activity in the prefrontal cortex.

#### 3.1.2. Temporal Cortex Components Analysis

**Figure 6:**
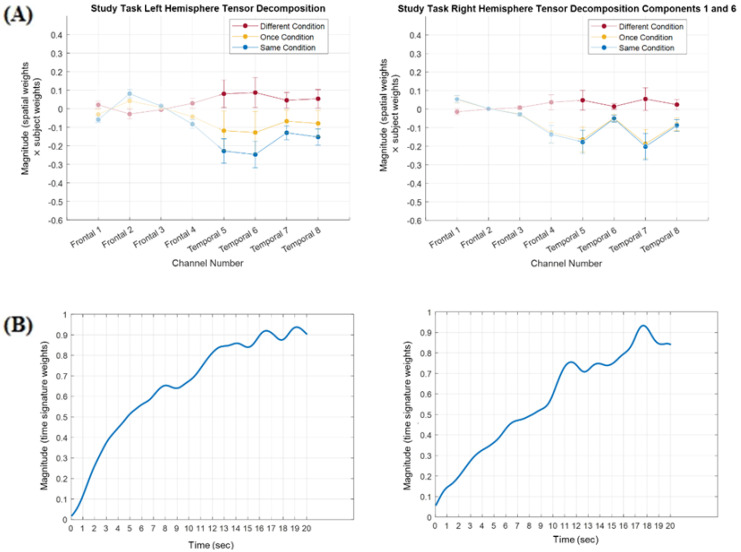
Left hemisphere temporal component 3 versus right hemisphere temporal component 1/6. (Panel **A**) In each component, the subject weights were multiplied by the spatial (channel) weight to obtain the values displayed in each channel. In order to highlight that these were temporal components, the frontal channels have been faded. (Panel **B**) The time modes for the left hemisphere component and the right hemisphere (combined) component. *N* = 48. Error bars: ±1 SE.

Based on the review of previous results with the HS-SC task and switch manipulations, another hypothesis was that the LATL and RATL play a role in representation, but not in selection. Based on this, we predicted that activity in the Different condition would NOT be greater than in the Same condition in either the LATL or RATL. Because both of the right hemisphere components displayed a Type of Processing effect in the temporal channels, they were combined into a single right hemisphere component (see Appendix B). Left hemisphere component 3, right hemisphere component 1/6, and their respective time modes are displayed in Figure 6. A 3 (Type of Processing) × 2 (hemisphere) × 4 (channels 5–8) repeated measures ANOVA was conducted on the subject × spatial weights of these components. There was a significant main effect of Type of Processing condition, *F*(1.77,83.07) = 4.22, *p* = 0.02, *MSE* = 1.25, *partial eta squared* = 0.08. Pairwise comparisons revealed that the Different condition (*M* = 0.05, *SE* = 0.05) was significantly greater than the Same condition (*M* = −0.16, *SE* = 0.05), *p* < 0.01, *d* = 0.50. The Once condition (*M* = −0.11, *SE* = 0.06) was marginally less than the Different condition, *p* = 0.06, *d* = 0.28. This result was not consistent with a priori predictions regarding hemodynamic activity in the temporal cortex and will be addressed in the discussion.

### 3.2. Comparing Tensor Decomposition and Grand Averaging

**Figure 7:**
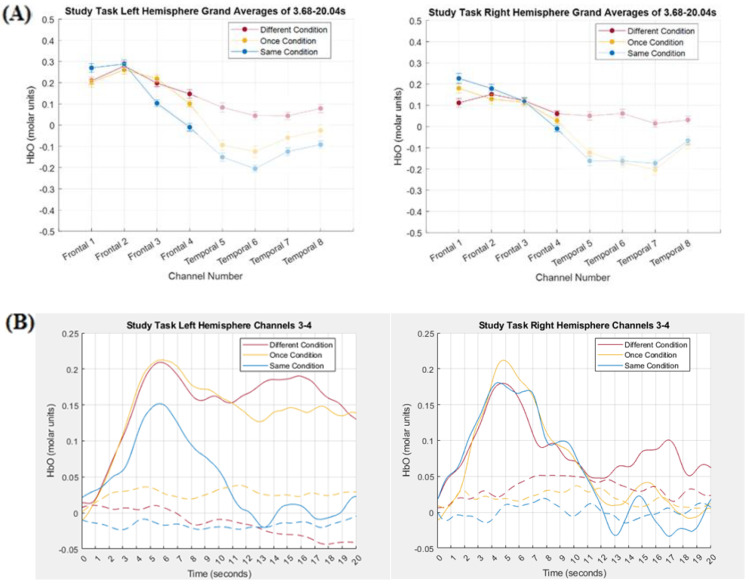
(Panel **A**) Grand averages for left and right hemispheres. In order to highlight that these grand averages were based on the TOIs for the frontal components, the temporal channels have been faded. (Panel **B**) HbO (solid) and HbR (dashed) hemodynamic response functions collapsed across channels 3 and 4.

**Figure 8:**
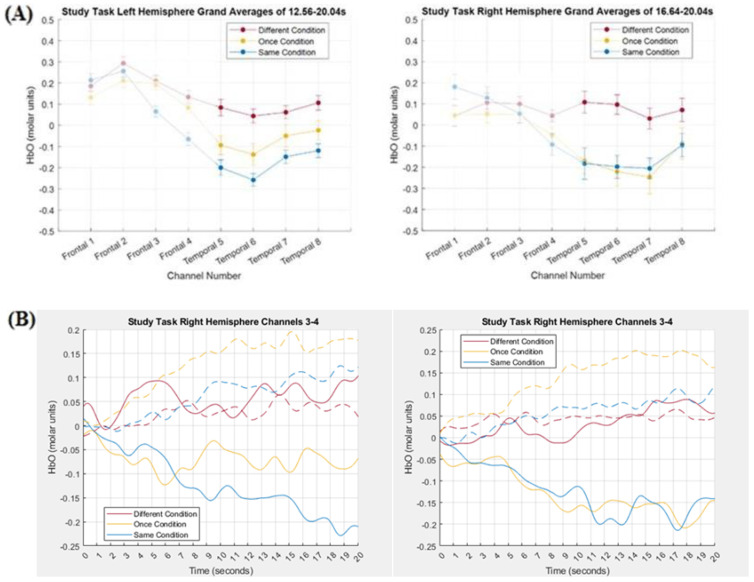
(Panel **A**) Grand averages for left and right hemispheres. In order to highlight that these grand averages were based on the TOIs for the temporal components, the frontal channels have been faded. (Panel **B**) HbO (solid) and HbR (dashed) hemodynamic response functions collapsed across channels 5–8.

In order to compare the TD results depicted in Figure 5 and Figure 6 with the grand averaging results, HbO magnitude was averaged across time but not across channels. A TOI was determined in the time mode of the components and then that TOI was used in the grand averaging. In order to determine a TOI in the time mode of the components, all contiguous values within −0.1 of the peak value were included in the TOI. In addition, if the weights fell below −0.1, but not below −0.2, and then increased again to greater than −0.1, those additional time points were also included in the TOI, as long as the length of time that the weights fell in the −0.1 to −0.2 range did not last for more than 3 s. Using this method, the TOIs of the time modes were 3.68–20.04 s for left hemisphere frontal component 1 and right hemisphere frontal component 2, 12.56–20.04 s for left hemisphere temporal component 3, and 16.64–20.04 s for right hemisphere temporal component 1/6. The HbO grand averages for the left and right hemisphere frontal channels are displayed in Figure 7 (Panel A) along with the hemodynamic response curves (Panel B), collapsed across channels 3 and 4, for both HbO and HbR in the left and right hemispheres. The HbO grand averages for the left and right hemisphere temporal channels are displayed in Figure 8 (Panel A) along with the hemodynamic response curves (Panel B), collapsed across channels 5–8, for both HbO and HbR in the left and right hemispheres.

TD can be conceptualized as a principal component analysis of multidimensional data. As such, it can be expected to produce components that are both independent of each other and represent a part of, but not the whole, hemodynamic response. For example, visual inspection of Figure 5 and Figure 7 revealed that left hemisphere component 1 and right hemisphere component 2 captured the relationship between the Different and Same conditions in frontal channels 3 and 4 in the grand average, but not the relationship between those conditions in channels 5–8 in the grand average. In contrast, visual inspection of Figure 6 and Figure 8 revealed that left hemisphere component 2 and right hemisphere component 1/6 captured the relationship between the Different and Same conditions in temporal channels 5–8 in the grand average, but not the relationship between those conditions in frontal channels 3 and 4 in the grand average.

These observations were supported statistically: Pairwise comparisons between the Type of Processing conditions in the grand averaging produced the same pattern of results as in TD. In left frontal channels 3 and 4, pairwise comparisons revealed that the Same condition (*M* = 0.05, *SE* = 0.05) was again less than the Different condition (*M* = 0.17, *SE* = 0.05), *p* = 0.03, *d* = 0.32 while, in right frontal channels 3 and 4, there again was no difference between the Same condition (*M* = 0.06, *SE* = 0.05) and the Different condition (*M* = 0.09, *SE* = 0.03), *p* > 0.47. Likewise, in bilateral temporal channels 5–8, pairwise comparisons revealed that the Different condition (*M* = 0.07, *SE* = 0.06) was again statistically greater than the Same condition (*M* = −0.18, *SE* = 0.06, *p* < 0.01, *d* = 0.50), while the Once condition (*M* = −0.12, *SE* = 0.08) was marginally less than the Different condition (*p* = 0.08, *d* = 0.26).

**Figure 9:**
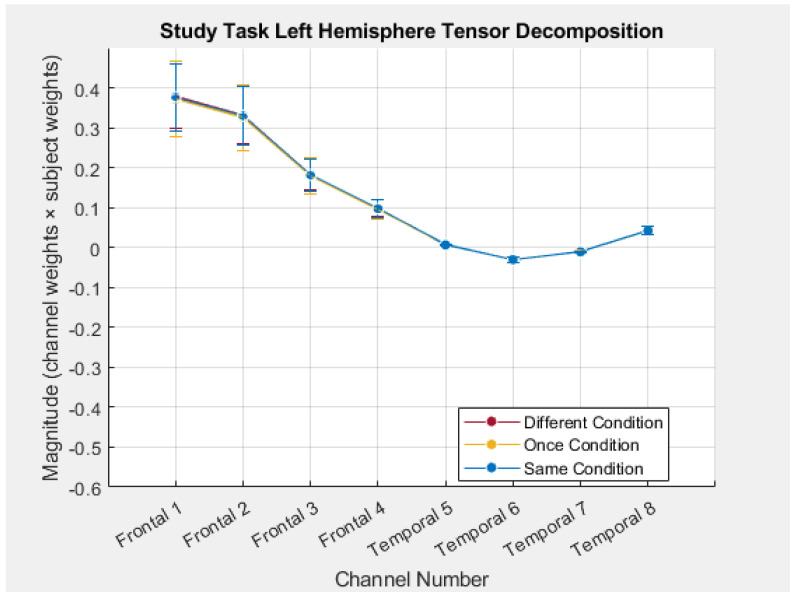
The subject weights were multiplied by the spatial (channel) weights to obtain the values displayed in each channel. *N* = 48. Error bars: ±1 SE.

As noted earlier, the main goal in using TD was to identify the spatial and time signatures of the Type of Processing effect (i.e., the differences between the Same, Different, and Once conditions). However, it should be noted that other components may highlight aspects of the hemodynamic response, other than the Type of Processing effect, that may also be important to understanding the association between hemodynamic response and task performance. For example, the subject × spatial weights plot for left hemisphere component 2 is displayed in Figure 9. A visual comparison of this plot with the grand average plot for the left hemisphere in Figure 8 revealed that left hemisphere component 2 captured the difference between relatively high levels of hemodynamic activity in the left frontal channels during the HS-SC task and relatively low levels of activation and deactivation in the left temporal channels during the HS-SC task, but not the differences between the Type of Processing conditions that were captured in other components. Again, this illustrated the fact that the components extracted by the TD approach were largely independent of each other and that each component represented part of, but not the whole, hemodynamic response. An interpretation of the pattern of hemodynamic activity captured in Figure 9 will be offered in the discussion.

### 3.3. Behavioral Results

Both the accuracy and response time (RT) results for the study phase are presented in Table 1. A one-way repeated measures ANOVA of accuracy revealed a significant effect of Type of Processing on accuracy, *F*(2,94) = 4.43, *p* = 0.02, *MSE* = 0.01, *partial eta squared* = 0.09. Pairwise comparisons showed greater accuracy in the Same condition (*M* = 0.85, *SD* = 0.09) compared to the Different condition (*M* = 0.78, *SD* = 0.12), *p* = 0.01, *d* = 0.43. Same condition accuracy was also marginally greater than in the Once condition (*M* = 0.80, *SD* = 0.14), *p* = 0.06, *d* = 0.42. A one-way repeated measures ANOVA on RT also revealed a significant main effect of Type of Processing, *F*(2,94) = 34.27, *p* < 0.01, *MSE* = 17,964.02, *partial eta squared* = 0.42. Pairwise comparisons showed significant differences between all three Types of Processing conditions; all *p*’s < 0.01. The Once condition had the slowest RT (*M* = 1553.51, *SD* = 177.69), followed by the Different condition (*M* = 1460.27, *SD* = 167.93), and the Same condition (*M* = 1328.11, *SD* = 158.20). In short, at the behavioral level, there was evidence of priming in the Same condition in that accuracy was (marginally) greater and RT was significantly less than in the Once condition. In contrast, there was evidence of interference in the Different condition. That is, despite the fact that all three items in the triplet repeated in the Different condition (RT in the Different condition was significantly less than in the Once condition), Different accuracy was not greater than Once accuracy, Different accuracy was less than Same accuracy, and Different RT was greater than Same RT. Again, this pattern of results supported the notion that priming (and decreased demands on the selection process) occurred in the Same condition, while interference from the first cue dimension on the second (different) cue dimension occurred in the Different condition and increased demands on the selection process associated with the second cue dimension.

## 4. Discussion

The primary goal of the present experiment was to evaluate the role of the LIFG and LATL in the selection of conceptual knowledge from semantic memory. In order to instate a high degree of selection demands, a HS-SC task and a switch manipulation (in the Different condition)—both of which had been independently shown to increase selection demands relative to control conditions—were combined in the present experiment.

### 4.1. Synopsis

As expected, greater activation in the LIFG was observed in the Different condition (used to instate the switch manipulation in the HS-SC task) than in the Same condition (in which the HS-SC of particular brand-name products was repeated). This was observed in the more ventral and posterior channels 3 and 4, which corresponded to Brodmann’s areas 44 and 45, which together are classically considered to be Broca’s area. This same Type of Processing difference was not observed in the more dorsal and anterior channels 1 and 2, which corresponded to Brodmann’s area 46, which is often associated with working memory function. No differences between those conditions were observed in the RIFG. The contrast between the patterns of activation in the LIFG and RIFG supported the notion that the LIFG plays a role in the selection of a concept from semantic memory when the retrieval of that concept is specific to a particular context. In addition, there were three other aspects of the Type of Processing effect that proved interesting.

First, in channels 3 and 4, the level of activation in the Different condition was equivalent to the level of activation in the Once condition. This observation was contrary to the expectation that the Different condition in the HS-SC task would produce greater relative activation than in the Once condition because of higher selection demands.

Second, in channels 3 and 4, the level of activation in the Same condition was less than in the Once condition. Often, repetition produces activity levels below zero, often referred to as repetition suppression [35]. However, while there was some evidence for repetition suppression in channels 3 and 4 in the subject x spatial weight plots, there was very little evidence for repetition suppression in those channels in the grand averaging plots. Thus, we would argue that the differences in activation levels between the Same and Once conditions in channels 3 and 4 were consistent with the notion that repetition facilitated selection, but the differences are probably better characterized as relative deactivation in the Same condition, rather than repetition suppression, per se.

Third, in the bilateral ATL and bilateral superior temporal gyrus (STG), greater activation in the Different condition than in the Same condition was observed. Moreover, in those temporal channels, activation in the Different condition was marginally significantly greater than in the Once condition. Although there have been occasional reports of ATL and STG activity in some switch tasks, no differences in activity due to selection demands have been reported in those regions [2,36,37]. The next section discusses how these unexpected findings might be explained by reexamining the processing demands levied by the Type of Processing manipulation used here.

### 4.2. Potential Reasons for Unusual Findings

#### 4.2.1. Lack of Selection Demands?

One possible explanation for the absence of greater activation in the Different condition than in the Once condition in the LIFG was that the Different condition in the HS-SC task did not actually engender different processing than the Once condition in the HS-SC task. However, two results suggested that this was not the case. First, behaviorally, response time in the Different condition was significantly faster than in the Once condition. Second, temporal lobe activation in the Different condition was greater than in the Once condition.

Another possible explanation for the absence of greater activation in the Different condition than in the Once condition in the LIFG was that selection demands were actually less than in previous experiments. However, besides the fact that the combination of the HS-SC task and the switch condition should have instated a higher degree of selection demands than in previous experiments, many more cue dimensions were used in the present experiment and those cue dimensions varied from behavioral trial to behavioral trial, forcing participants to select a different dimension (and a different value) on each trial. Hypothetically, both of these differences should also have increased selection demands relative to how the HS-SC task has been operationalized in past experiments c.f., [15,38].

Of course, one possibility was that post-retrieval selection, as instated in the combination of the HS-SC task and the Different condition used here, was actually the purview of temporal lobe regions, rather than the LIFG. However, given the overwhelming evidence in prior work that the locus of selection is the LIFG, we are reluctant to posit that temporal regions were instead the locus of selection in this particular experiment. Rather, despite all the factors listed above that suggest that the combination of the HS-SC task and the Different condition, at least on the face of it, should have instated a high degree of selection demands, it seems likely now that other elements may have been instated in the manipulation that, even though they were successful in producing significant differences between the conditions, shifted the differences from the LIFG to temporal lobe regions. With this in mind, some potential reasons for the shift of the Type of Processing effect—especially the difference between the Different and Once conditions—from its expected locus in the LIFG to bilateral ATL and STG are offered next.

#### 4.2.2. Stimulus Characteristics?

One possible reason for the greater sensitivity of temporal regions rather than the LIFG to the difference between the Different and Once conditions might be the use of brand-name products in the present experiment rather than common nouns. Although the retrieval and comprehension of proper nouns can be dissociated from that of common nouns [39], the research is mixed as to the locus of proper noun processing. On the one hand, the names of people and brands are sensitive to a semantic access deficit, which in turn implies the involvement of the LIFG in the processing of both people names and brand names [40,41]. On the other hand, the left temporal pole is involved in retrieving a name when presented with “semantically unique” entities, such as landmarks, faces, voices, and melodies [42], and in retrieving conceptual information for such entities when presented with a name [43]. Although proponents of the notion that the LIFG is the locus of semantic control have often argued that it is a domain-general mechanism [44], perhaps proper nouns are an exception to this claim. After all, the association of a proper name with a unique entity may not be as much a problem of selection from amongst multiple viable alternatives that have been retrieved from semantic memory as much as it is a problem of searching through and locating the proper name of that unique entity in the semantic memory store itself.

Another possible reason for the greater sensitivity of temporal regions rather than the LIFG to the difference between the Different and Once conditions might be the use of location information in the present experiment. For example, using multivoxel pattern analysis of fMRI, Peelen and Caramazza [45] found that the difference between objects typically found in a garage location and objects typically found in a kitchen location was computed in the ATL. There were two ways in which location had a larger presence in the present experiment than in past semantic comparison experiments: “setting” was used as a cue dimension in 23 of the 96 triplets and pictures of businesses were used in another 14 of the 96 triplets. Perhaps the inclusion of location information elicited greater ATL involvement than in prior semantic comparison experiments in which location information did not play a role.

#### 4.2.3. Alternative Conceptualizations of the High-Selection Semantic Comparison Task?

Another possible explanation for the pattern of results associated with the Different condition was that the Different condition was not so much an operationalization of a high degree of selection, but was more an operationalization of flexible retrieval. Flexible retrieval is the retrieval of a concept that is specific to a particular context, such that, as context varies over time, so does processing of the concept, much like the car example described in the opening paragraph, or the Elmer’s Glue example referred to throughout this paper. In their review of the neural basis of contextually-sensitive conceptual retrieval, Folstein and Dieciuc [46] noted that the LIFG, along with temporoparietal regions, plays a primary role in the context-sensitive processing of concepts [47,48]. However, if the LIFG was the locus of flexible retrieval in the present experiment, then one would have expected greater activation in the Different condition, where the context varied over time, than in the Once condition, where selection specific to a particular context was needed, but did not vary over time. Of course, in the present experiment, activation in the Different condition was equal to, not greater than, activation in the Once condition. This result supported the notion that, while the LIFG played a role in selection, it did not play a direct role in the type of flexible retrieval of semantic information that may have been instantiated in the Different condition in the present experiment.

Finally, another possibility was that the HS-SC task was also an operationalization of the construction of ad hoc categories [49], in addition to an operationalization of selection. An ad hoc category is a situation-specific category (e.g., things that can be worn on the legs on a cool evening) that is constructed online (not retrieved from long-term semantic memory) and cuts across a variety of common nominal categories (e.g., blankets, sweatpants, leggings, jeans, etc.). For example, if the situational information presented was “Color” (as shown in Figure 2), then the participant needed to create an ad hoc category in which two of the three products (Elmer’s Glue and Imperial Sugar) shared the same color (i.e., white) and belonged to the same ad hoc category (i.e., things that are white).

There is some evidence that supports the notion that the construction of ad hoc categories is probably the purview of the LIFG [46]. For example, Corbett et al. [50] found that semantic aphasia patients (damage to frontoparietal areas associated with semantic control) were significantly better at selecting a fly swatter from an array of objects in order to kill a fly than they were at selecting a magazine from an array of objects (that did not include a fly swatter) in order to kill a fly. There is also some evidence that the construction of ad hoc categories is not the purview of the ATL [46]. For example, Peelen and Caramazza [45] found that, while the difference between objects typically found in a garage and objects typically found in a kitchen was computed in the ATL, this computation was not modulated by whether the task demand was to differentiate between the typical locations of the objects or the typical operations of the objects.

Nevertheless, given the dearth of research exploring the neural underpinnings of ad hoc category constructions, Folstein and Dieciuc [46] left open the possibility that the ATL could play a role in the construction of ad hoc categories. After all, when such categories have been constructed on a regular basis in order to meet an oft-experienced, situation-specific need or goal (e.g., warming one’s legs), they are referred to as goal-derived categories [51]. Such categories become well-established in memory—probably in the ATL—and this leaves open the possibility that even early ad hoc iterations of these goal-derived categories could be constructed in the ATL. If indeed the high-selection semantic comparison task includes elements of ad hoc category construction and if ad hoc category construction is indeed the purview of the ATL, then one might expect relative deactivation in the ATL (compared to the Once condition) when ad hoc category construction is repeated (in the Same condition), and relatively heightened activation in the ATL (compared to the Once condition) when the ad hoc category is switched (in the Different condition). The results of the present experiment were largely consistent with these post-hoc predictions and, as such, provided support for the notion that ad hoc category construction is indeed the purview of the ATL. Of course, this after-the-fact claim is speculative and it is our hope that current research in our lab will shed further light on both the nature of ad hoc categories and their neural underpinnings.

### 4.3. Evaluation of the Tensor Decomposition Approach to fNIRS Data Analysis

The main goal of using TD was to identify the spatial and time signatures of the Type of Processing effect (i.e., the differences between the Same, Different, and Once conditions). Using TD altered our view and interpretation of the hemodynamic response in a few important ways. First, instead of operating on the assumption that there would be a frontocortical ROI that was distinct from a temporocortical ROI, especially in the left hemisphere, the components that emerged from the atheoretical TD clearly indicated the distinction between those ROIs not only in the left hemisphere, but also in the right hemisphere. Second, instead of operating on the assumption that all four left frontal channels would form an ROI, the frontal component that emerged from the left hemisphere TD clearly indicated that only channels 3 and 4 were sensitive to the Same–Different distinction. Third, our initial attempts to establish a TOI for the frontocortical ROI proved to be difficult because it assumed that the ROI included all four channels and relied on visual inspection of the hemodynamic response functions for all three conditions. Instead, the time mode for a particular component is represented in a single function when the subject mode of that component (that particular difference between conditions) and the spatial mode of that component (that particular pattern of activation across the channels) are making their peak contributions to that particular component. The fact that all of this is represented in a single time mode function made it a more straightforward matter to establish a relatively simple rule for establishing the TOIs. Fourth, even though our emphasis was on identifying the spatial and time signatures of the Type of Processing effect, other components that did not capture the Type of Processing effect—such as left hemisphere component 2—can direct our attention to other aspects of the hemodynamic signal that may also aid in understanding the role that different ROIs play in task performance. For example, the difference between the levels of hemodynamic activity in the LIFG and LATL supported the notion that these two ROIs indeed played different roles in the HS-SC task. Assuming for the moment that semantic control is the purview of the LIFG and semantic representation is the purview of the ATL, then this observation could indicate that the demands on cognitive resources for semantic control were greater than those for semantic representation in the HS-SC task. Nevertheless, even though the resource demands on semantic representation were relatively low, it was still the case that switching the cue dimension and the relationship between those concepts in the Different condition placed a greater demand on those resources in the bilateral ATL and STG than did the Once and Same conditions (c.f., [52]).

Given that the TD approach to analyzing fNIRS data is so new and computationally dense, it seemed important to validate the products of the approach by comparing those products to the products of the more familiar grand averaging approach, when the ROIs and TOIs indicated in the TD were used to compute the grand averages. When the grand averages for each ROI were computed and the differences between conditions in those grand averages were compared to the differences between conditions in the subject weight x spatial weight plots, those differences corresponded across the TD and grand averaging approaches in all four ROIs. This outcome should enhance confidence that analysis of the components themselves, without reference to grand averages, can provide a valid picture of the fNIRS hemodynamic response. Nevertheless, at this early stage of applying TD to fNIRS data analysis, we still see value in using the correspondences between both approaches to drive theoretical conclusions.

## 5. Limitations and Future Directions

Several limitations and potential alterations to the current study can be noted. For example, there was a great deal of variability in the types of products and in the relationships between those products in the current experiment. Perhaps incorporating a more narrow focus on these elements of the materials would reduce statistical error variability and increase power in future experiments.

There were also fNIRS limitations that were specific to the current experiment. First, our optode configuration did not include more dorsal areas of the prefrontal cortex that are known to be important for cognitive control, sensory cortices known to be activated during the retrieval of semantic knowledge (i.e., the spokes in the hub-and-spokes model [3]), or temporoparietal areas known to be associated with semantic control. Second, using a block design limited the ability to adequately explore the correlations between hemodynamic activity and behavior, because neither is tied to the presentation of individual trials. Third, short separation fibers to remove noise in the hemodynamic signal associated with superficial layers of the head were not used, although it does not appear that the lack of using them prevented the detection of hemodynamic activation as a result of our experimental manipulations, at least in this particular experiment.

Finally, there were also TD limitations that were specific to the current experiment. First, in the current experiment, separate tensors were constructed for each hemisphere. However, four separate tensors could have been created, one for each ROI (i.e., LF, LT, RF, and RT), or two separate tensors could have been constructed, one for each lobe across hemispheres, or one tensor could have been constructed for the entire configuration of our optodes (i.e., all four ROIs in one tensor). There are pros and cons to each of these approaches that could be explored in future research. Second, despite our best efforts to use a quantitative basis for all of our analytic decisions, we were still left with using visual inspection in order to determine the ROI in each separate subject x spatial representation of the components that had significant Type of Processing effects (see results of Figure 5). Third, we used cluster analysis to choose a single run whose components were closest to the centroids. Any single run can fully represent the hemodynamic signal by summing across all of the components in that run. However, if the emphasis was instead on the larger components, like it was in the present experiment, then an alternative approach could be to select the larger components that are similar and that consistently occur across multiple runs and to use composite representations of those components as the basis for interpreting the hemodynamic response.

## 6. Conclusions

The use of tensor decomposition to analyze fNIRS output provided important, perhaps even indispensable, contributions to our understanding and explanations of the patterns of hemodynamic activity associated with flexible retrieval from semantic memory. Whether the flexible retrieval of semantic information in the high-selection semantic comparison task is conceptualized as switching ad hoc categories or not, flexible retrieval of semantic information in this experiment was associated more with bilateral temporal regions than with the left prefrontal cortex. This could be due to the use of proper nouns (i.e., brand-name products), rather than common nouns. As such, the combination of task and stimuli used in the present experiment may provide, in future research, the opportunity to probe temporal lobe contributions to semantic cognition that have not been previously explored.

## Figures and Tables

**Table 1:** Behavioral results.

	Type of Processing		
	Once	Same	Different
Response time	1554 (178)	1328 (158)	1460 (168)
Accuracy	0.80 (0.15)	0.85 (0.09)	0.78 (0.12)

Note. Response time is in ms. Means are bolded. SDs are in parentheses. *N* = 48.

## Data Availability

The raw data supporting the conclusions of this article will be made available by the authors upon request. The data are not publicly available because consent to publicly share the data was not obtained through informed consent at the time the data was collected.

## Supplementary Materials

The following supporting information can be downloaded at: https://www.mdpi.com/article/10.3390/brainsci15020127/s1, Figure S1. A magnified version of the third slide in the example of the time course of a behavioral trial displayed in Figure 2; Figure S2: Average optode location digitization associated with the placement of the fNIRS headgear; Figure S3: Tensor construction and decomposition; Figure S4: Pipeline for the preprocessing of the fNIRS signal (in blue), tensor decomposition (in green), and validation of tensor decomposition with grand averaging (in orange); Figure S5. A magnified version of Figure 4, subfigure (C) – the spatial mode weights of left hemisphere component 1; Table S1: Order of Type of Processing conditions in study phase; Table S2: The 96 stimulus sextuplets; Table S3. MNI coordinates of the average optodes placement.

## Appendix A

### Appendix A.1. Tensor Construction

A three-way tensor with time × spatial × subject modes (X $\in I_{t}\times I_{c}\times I_{s}$) was constructed for the study phase data. $I_{t\;}$ represents the number of time samples, $I_{c\;}$ represents the number of channels, and $I_{s\;}$ represents the number of subjects x conditions. Given that the current study was a within-subjects design, to calculate the total number of subjects in the subject mode would be to multiply the number of subjects with the number of conditions. For example, there were 48 participants and 3 conditions in the study task, making 144 subjects in the subject mode.

### Appendix A.2. Tensor Decomposition

CPD with an orthogonal constraint on the spatial mode was used to decompose each three-way tensor X into R number of rank-1 components for the time mode$\;u_{r}^{\left(t\right)}$, spatial mode $u_{r}^{\left(c\right)}$, and subject mode $u_{r}^{\left(s\right)}$. Each component was constructed by multiplying a vector from the time mode, spatial mode, and subject mode (see Equation (A1)). For example, the first component (r = 1) consisted of multiplying the first-time vector $u_{1}^{\left(t\right)}$, with the first spatial vector $u_{1}^{\left(c\right)}$, with the first subject vector $u_{1}^{\left(s\right)}$.

(A1) $$\underline{X}\;\approx \;\sum_{r=1}^{R}u_{r}^{\left(t\right)}{\cdot u}_{r}^{\left(c\right)}{\cdot u}_{r}^{\left(s\right)}$$

Orthogonal constraints were used to ensure a more unique decomposition [53]. The alternating least squares (ALS) algorithm was used to perform the CPD. This procedure was performed for 10 runs.

## Appendix B

### Determination of TOI and ROI

The corresponding significant time modes $u_{r}^{\left(t\right)}$ and spatial modes $u_{r}^{\left(c\right)}$ multiplied by subject modes $u_{r}^{\left(s\right)}$ that show similar patterns were selected and weighted in proportion to the percentage of the signal they accounted for P and divided by the number of selected components in the according condition and hemisphere to reveal the time mode profile and spatial by subject weights of the significant difference. Z was the list of the selected significant components from the according task and hemisphere. If the first and second components revealed a significant effect and had similar patterns, then Z = {1, 2}. P is the percentage that the component accounted for in the original tensor X. Equation (A2) demonstrates how the time mode profile was constructed. Equation (A3) demonstrates how the spatial weights × the subject weights was constructed. If there were multiple components that were statistically significant, similar components were selected to be used for interpretation.

(A2) $$\mathrm{Time}\;\mathrm{mode}\;\mathrm{profile}\;\mathrm{for}\;\mathrm{multiple}\;\mathrm{components}=\frac{\sum_{r\in Z}u_{r}^{\left(t\right)}\times P}{\sum_{r\in Z}P}$$

(A3) $$\mathrm{Spatial}\;\mathrm{by}\;\mathrm{subject}\;\mathrm{weights}\;\mathrm{for}\;\mathrm{multiple}\;\mathrm{components}=\frac{\sum_{r\in Z}u_{r}^{\left(c\right)}\;{\cdot u}_{r}^{\left(s\right)}\times P\;}{\sum_{r\in Z}P}$$

In the case where there were no similar emerging components to combine, the time mode profile was constructed by just taking the time mode $u_{r}^{\left(c\right)}$ weights. Equation (A4) demonstrates how the time mode profile was created for one component. The weights from the spatial modes $u_{r}^{\left(c\right)}$ were multiplied by the weights from the subject modes $u_{r}^{\left(s\right)}$ to produce the spatial weights × subject weights profiles. Equation (A5) demonstrates how the spatial weights × the subject weights were constructed for just one component.

(A4) $$\mathrm{Time}\;\mathrm{signature}\;\mathrm{profile}\;\mathrm{for}\;\mathrm{one}\;\mathrm{component}=u_{r}^{\left(t\right)}$$

(A5) $$\mathrm{Spatial}\;\mathrm{by}\;\mathrm{subject}\;\mathrm{weights}\;\mathrm{for}\;\mathrm{one}\;\mathrm{component}=u_{r}^{\left(c\right)}{\cdot u}_{r}^{\left(s\right)}$$

In the event that there was not a statistically significant component, the component that was marginally significant was selected to be used for interpretation.

It is important to note that the time mode profile is not an HRF. The time mode profile represents the culmination of significant patterns revealed with CPD and ANOVA. The peak in the time mode profile marks the TOI during which the significant differences between conditions revealed by CPD and ANOVA are the most prominent. The points of time with lower values in the time profile signature indicate little differences across conditions.

The spatial weight × subject weight values indicated the channel(s) that revealed big differences across conditions. Channels with the highest or lowest values indicated the ROI that revealed the biggest differences across conditions. Channels that were neighboring to the one with the highest or lowest magnitude were grouped into an ROI. Channels with values close to zero indicated little differences across conditions.
