# Supplementary material for: Effects of Competition on Left Prefrontal and Temporal Cortex During Conceptual Comparison of Brand-Name Product Pictures: Analysis of fNIRS Using Tensor Decomposition
Source: Brain Sci. 2025 Jan 28;15(2):127. doi: 10.3390/brainsci15020127 (PMC11852890; doi:10.3390/brainsci15020127)

brain sciences

MDPI

Table S1. Order of Type of Processing conditions in study phase.

fNIRS Block	Conditions for all Participants
1	Oncea
2	Sam1a
3	Diff1a
4	Sam1b
5	Diff1b
6	Onceb
7	Diff2b
8	Sam2b
9	Diff2a
10	Sam2a
11	Rest
12	Oncec
13	Diff1c
14	Sam1c
15	Diff1d
16	Sam1d
17	Onced
18	Sam2d
19	Diff2d
20	Sam2c
21	Diff2c

Note. The data for each condition that was entered into both the tensor decomposition and the grand average analyses was the average hemodynamic response across the four fNIRS blocks in that condition. For example, performance in the Different condition was obtained by averaging across the four Diff2 blocks (a, b, c, and d) for each participant.

Table S2. The 96 stimulus sextuplets

Cue product 1	Cue product 2	Target product 1	Target product 1 cue dimension	Target product 2	Target product 2 cue dimension
Callaway Golf Shoes	FootJoy Golf Shoes	Steve Madden Heels	Body part	Wilson Tennis Racket	Leisure activity
Revlon Nail Polish	Essie Nail Polish	Clinique Lipstick	Color	Sally Hansen Nail Clipper	Body Part
London Fog Umbrella	Totes Umbrella	Hot Tools Curling Iron	Shape	Hunter Rain Boots	Purpose
Good Housekeeping Magazine	Better Homes and Gardens Magazine	Seventeen Magazine	Gender	Wall Street Journal	Age of Reader
Johnnie Walker Scotch	Chivas Regal Scotch	Keystone Beer	Color	Absolut Vodka	Alcohol Content
Korbel Champagne	Freixnet Champagne	Ozarka Natural Spring Water	State of matter	Petrossian Caviar	Price
Lazy-Boy Recliner	Powell Recliner	Whirlpool Dishwasher	Size	Coleman Camping Stool	Purpose
Kikkerland Paper Straws	Green Party Straws	Camel Cigarettes	Shape	Jamba Juice Fruit Smoothie	Purpose
L'eggs Leggings	No nonsense Leggings	Burberry Perfume	Gender	Dockers Dress Pants	Body Part
Paramount Pictures "The Godfather"	Warner Bros. "Goodfellas"	Rockstar Games "Grand Theft Auto"	Content	20th Century Fox "The Sound of Music"	Type of Media
San Jamar Dishwashing Gloves	Casabella Dishwashing Gloves	Palmolive Dish Detergent	Setting	Vibram Toe Shoes	Shape
Baby Jogger Stroller	Graco Jogger Stoller	Adidas Running Shorts	Leisure activity	Jackson Wheelbarrow	Function
Kobalt Screwdriver	Klein Tools Screwdriver	Papermate Pen	Shape	Craftsman Hand Saw	Setting
Altoids Mints	tic tac Mints	Colgate Toothpaste	Flavor	One-A-Day Vitamins	Size
Tuff Shed	Suncast Shed	Toyota Camry Automobile	Size	Scotts Fertilizer Spreader	Setting
Formula 409 All-Purpose Cleaner	Lysol All-Purpose Cleaner	Irish Spring Bar Soap	Purpose	Horizon Organic Milk	State of Matter
Brach's Lemon Drops	Lemonhead Lemon Candy	Sprite Soda	Flavor	Strawberry Fruit Roll-Ups	State of Matter

Dollar Tree	99 cents Only	Goodwill Store	Price	Lowe's Home Improvement	New/Used
Ann Taylor	Chico's	Abercrombie & Fitch	Variety of Goods	Jo-Ann Fabrics and Crafts	Gender
Houston Chronicle Newspaper	San Antonio Express Newspaper	Suddenlink Satellite/Cable Provider	Purpose	Solo Paper Cups	Composition
**Cue product 1**	**Cue product 2**	**Target product 1**	**Target product 1 cue dimension**	**Target product 2**	**Target product 2 cue dimension**
Haribo Gold-Bears Gummi Candy	Black Forest Gummy Bears	Trident Gum	Texture	Teddy Grahams Cracker Snacks	Shape
Braun Electric Shaver	Phillips Norelco Electric Shaver	Samsung Galaxy Phone	Size	Crane Toilet	Setting
Snyder's Pretzels	Rold Gold Pretzels	Ruffles Original Potato Chips	Food Department	Libby's Green Beans	Shape
Great Clips Salon	SuperCuts Salon	Zales Jewelry Store	Health and Beauty	Jason's Deli	Price
The North Face Backpack	Jansport Backpack	Louis Vuitton Purse	Size	Texas Instruments Calculator	Setting
Aqua Net Hair Spray	Paul Mitchell Hair Spray	L.A. Looks Hair Gel	Body Part	Febreze Air Effects Room Spray	State of matter
Saks Fifth Avenue	bloomingdale's	Kohl's	Variety of Goods	Mercedes-Benz Car Dealership	Price Range
Meads Spiral Notebook	Top Flight Spiral Notebook	MacBook Air Computer	Setting	Vanity Fair Napkins	Composition
Robitussin Cough Syrup	Mucinex Cough Syrup	Gordon's Gin	State of matter	Claritin Reditabs	Purpose
Dole Bananas	Del Monte Bananas	Ocean Spray Apple Juice	Food Group	Sara Lee Bread	State of matter
Grip-Rite Nails	Maze Nails	Falk Copper Pots	Composition	Ticonderoga Woodcase Pencil	Shape
Bass Pro Shop	Cabela's	AutoZone Auto Parts	Gender	Sam's Club	Size
Visine Eye Drops	Systane Eye Drops	Ray Ban Sunglasses	Body Part	Aveeno Lotion	State of matter
Bicycle Playing Cards	Bee Playing Cards	Xbox One Game Console	Leisure activity	Reader's Digest Magazine	Weight

Polder Drying Rack	Honey-Can-Do Drying Rack	Snuggle Fabric Softener	Setting	SWEPCO Electric Company Powerlines	Structure
Cavender's Cowboy Hat	Justin Cowboy Hat	JVC Headphones	Body part	Anvil Horseshoe	Setting
Cinemark Movie Theatre	AMC Movie Theater	Starbucks Coffee Store	Cost	NEC Multimedia Digital Projector	Purpose
GUM Soft Picks	Doctor's Brush Picks	Crest Mouthwash	Body Part	John James Sewing Needles	Shape
Mad River Canoe	Clipper Canoe	Schwinn Bike	Mode of Transportation	Bayliner Boat	Exercise
Goody Hair Comb	Conair Hair Comb	Suave Shampoo	Body Part	Dixie Plastic Fork	Structure
**Cue product 1**	**Cue product 2**	**Target product 1**	**Target product 1 cue dimension**	**Target product 2**	**Target product 2 cue dimension**
Promised Land Chocolate Milk	TruMoo Chocolate Milk	Lipton Iced Tea	State of matter	Duncan Hines Chocolate Brownie Mix	Ingredients
Farm Patch Farmers' Market	Brazos Valley Farmers' Market	HEB	Type of goods	Milberger's Plant Nursery	Setting
Target Retail Store	Wal-Mart Retail Store	CVS Pharmacy	Type of goods	Home Depot	Size
Georgia Pacific Copy Paper	Hammermill Copy Paper	Toshiba TV	Shape	Wham-O Ultimate Frisbee	Size
Ghirardelli Chocolate Chips	Hershey's Chocolate Chips	Milky Way Candy Bar	Flavor	Universal Push Pins	Size of Contents
SanDisk USB Flash Drive	Lexar USB Flash Drive	Asus ZenPad Tablet	Type of Product	Pentel Hi-Polymer Eraser	Size
Kammok Hammock	ENO Hammock	Serta Mattress	Purpose	Wild Birds Unlimited Bird Feeder	Setting
Tommy Hilfiger Billfold	Tumi Billfold	Canon Camera	Size	Tony Lama Cowboy Boots	Composition
Kaytee Bird Food	HigginS Bird Food	Iams Dog Food	Purpose	McCormick Yellow Mustard Seed	Size of Contents

Shipley's Donuts	Krispy Kreme Donuts	Trix Cereal	Shape	Borden's American Cheese Slices	Size
Odor-Eaters Foot Powder	Dr Scholl's Foot Powder	Degree Deodorant	Purpose	L'Oreal Face Powder	State of matter
Pampers Diapers	Huggies Diapers	Quilted Northern Toilet Paper	Body Part	Sassy Baby Rattle	Age of User
Spotify Internet Radio	Pandora Internet Radio	Hulu Plus	Type of Access	Beyonce Concert	Type of Media
Samsonite Suitcase	Travelpro Suitcase	Husky Trash Bags	Capacity	Calvin Klein Men's Toiletry Bag	Purpose
Louisville Slugger Baseball Bat	Marucci Baseball Bat	Remington .22 Caliber Rifle	Size	Voit Volleyball	Leisure activity
Blue Rhino Propane Tank	BernzOmatic Propane Tank	Duracell Batteries	Shape	Igloo Ice Chest	Size
Black && Decker Leaf Blower	Echo Leaf Blower	Oreck Vacuum Cleaner	Purpose	True Temper Shovel	Setting
Shell Gas Station	Exxon Gas Station	Sonic Drive-In	Ease of Use	Maaco Auto Body Repair	Type of product
**Cue product 1**	**Cue product 2**	**Target product 1**	**Target product 1 cue dimension**	**Target product 2**	**Target product 2 cue dimension**
HON File Cabinet	Office Designs File Cabinet	Hewlett Packard Printer	Setting	Bassett Furniture Chest of Drawers	Purpose
CytoSport Muscle Milk Powder	BSN Syntha 6 Protein Powder	Powerade Sports Drink	Setting	Gold Medal All-Purpose Flour	State of Matter
International Delight Creamer	Nestle Coffee-mate Creamer	Sweet'N Low Sweetener	Purpose	Minute Maid Orange Juice	State of Matter
Mrs. Cubbison's Croutons	Reese Croutons	Wishbone Dressing	Purpose	Green Giant Niblets Corn	State of Matter
Sargento Swiss Cheese	Alpine Lace Swiss Cheese	Yoplait Light Yogurt	Food Group	Hillshire Farm Deli Ham	Purpose
Baldwin Piano	Bosendorfer Piano	Yamaha ATV	Size	Hohner Harmonica	Purpose

Post-it Sticky Notes	Quill Sticky Notes	Mastercard Credit Card	Size	USA Today Newspaper	Composition
Isokinetic, Inc. Exercise Ball	Go Fit Exercise Ball	24 Hour Fitness Center	Purpose	Cuties Clementines	Shape
Toys "Я" Us	Learning Express	Office Depot	Size of Store	Gymboree Children's Apparel	Age of Consumer
Royal Caribbean Cruise Lines	Carnival Cruise Lines	American Airlines	Size	Vanguard Sailboat	Mode of Transportation
Stihl Chainsaw	Husqvarna Chainsaw	Buck Pocket Knife	Purpose	Brunswick Bowling Ball	Weight
O-Cedar Broom	Libman Broom	Gibson Guitar	Shape	Harper Dustpan	Purpose
Frigidaire Refrigerator	LG Refrigerator	Rubbermaid Food Storage Containers	Purpose	Broyhill Sofa	Size
Roundup Weed Killer	Spectracide Weed Killer	Lawn-Boy Mower	Purpose	Penzoil Motor Oil	State of Matter
McFarlane Halo Action Figure	Bandai Dragonball Z Action Figure	Tonka Fire Truck	Gender	Mattel Barbie Doll	Shape
Fujitsu Image Scanner	Epson Image Scanner	Xerox Office Copier	Purpose	Panasonic DVD Player	Size
Hellman's Mayonaisse	Kraft Mayonnaise	French's Mustard	Food Department	Daisy Brand Cottage Cheese	Color
Sylvania Lightbulb	Cree Lightbulb	Maglite Flashlight	Function	Qualatex Balloon	Shape
Fiskar's Scissors	Gingher Scissors	Schick Blade Razor	Purpose	Victorio Kitchen Tongs	Shape
**Cue product 1**	**Cue product 2**	**Target product 1**	**Target product 1 cue dimension**	**Target product 2**	**Target product 2 cue dimension**
E-Z Up Canopy	Impact Canopy	Neutrogena Suncreen	Purpose	Pure Fun Backyard Trampoline	Size
Bengay Pain Relieving Cream	Thera-Gesic Pain Relieving Cream	Tylenol Pain Reliever	State of matter	Cetaphil Moisturizing Cream	Purpose
Golden Corral	Old Country Buffet	Cici's Pizza Buffet	Type of service	IHOP	Type of Food
Barnes & Noble	Hastings	Best Buy Store	Size	Bath and Body Works	Price

Kenmore Washing Machine	Haier Washing Machine	Cheer Laundry Detergent	Setting	Ameriwood Desk	Size
Libbey Wine Glass	Riedel Wine Glass	OXO Corkscrew	Setting	Ball Mason Jar	Composition
Swiffer Mop	Bona Mop	Brawny Paper Towels	Setting	Seymour Midwest Bow Rake	Shape
Pantene Hair Conditioner	Sunsilk Hair Conditioner	Heinz Ketchup	State of Matter	Body Benefits Bath Sponge	Setting
Hot Wheels Cars	Matchbox Cars	Hanes Boxer Briefs	Gender	Hasbro Easy Bake Oven	Age of User
Atrium Window	Cascade Window	Saran Plastic Wrap	Transparency	Lenoir Mirror	Composition
Favorite Findings Buttons	Haberdashery Buttons	Pepperidge Farm Cookies	Shape	Sullivans Zipper	Function
Logitech Computer Mouse	Lenovo Computer Mouse	JBL Speakers	Setting	Penn Tennis Ball	Size
Merona Scarf	Luxury Divas Scarf	Columbia Jacket	Season	Kendra Scott Necklace	Body Part
Giorgio Armani Briefcase	Hugo Boss Briefcase	DeWalt Toolbox	Purpose	Tommy Hilfiger Necktie	Setting
Hamilton Beach Blender	Oster Blender	Keurig Coffee Maker	Setting	BMW Motorcycle	Noise Level
Gutermann Thread	Mettler Thread	Glide Floss	Size	Singer Sewing Machine	Setting
Rusk Hair Dryer	Drybar Hair Dryer	Rowenta Clothes Iron	Temperature	Skintimate Shaving Cream	Setting
Pixar Animation Studios	Dreamworks Animation Studios	Six Flags Theme Park	Age of Consumer	NBC Studios	Type of Medium
**Cue product 1**	**Cue product 2**	**Target product 1**	**Target product 1 cue dimension**	**Target product 2**	**Target product 2 cue dimension**
Blue Cross Blue Shield Health Insurance	American Family Health Insurance	Geico Car Insurance	Type of service	Pfizer Pharmaceuticals	Setting

Table S3. MNI coordinates of the average optodes placement

Sources	Optode coordinates (MNI)
Left frontal (LF)	-34 42 9
Left temporal (LT)	-48 11 17
Right frontal (RF)	36 35 10
Right temporal (RT)	63 12 16
Detectors	
Left hemisphere	
LF1	-30 50 1
LF2	-38 49 -1
LF3	-28 36 -1
LF4 and LT5	-39 20 -2
LT6	-52 6 -14
LT7	-58 -1 -6
LT8	-53 -13 0
Right hemisphere	
RF1	38 46 1
RF2	48 49 -3
RF3	42 36 -1
RF4 and RT5	57 22 -6
RT6	54 5 -12
RT7	62 2 -5
RT8	49 -8 2

Figure S1. A magnified version of the third slide in the example of the time course of a behavioral trial displayed in Figure 2.

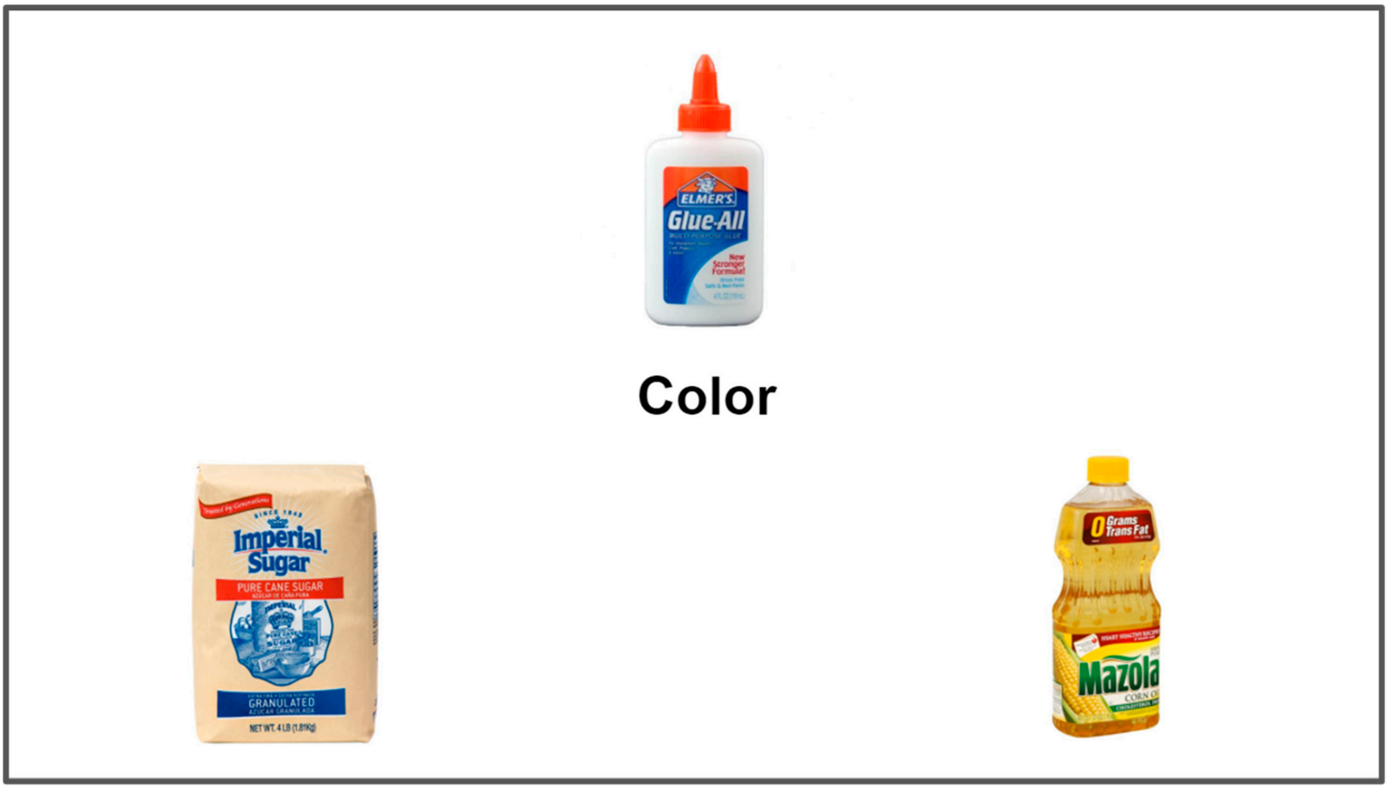

Figure S2. Average optode location digitization associated with the placement of the fNIRS headgear

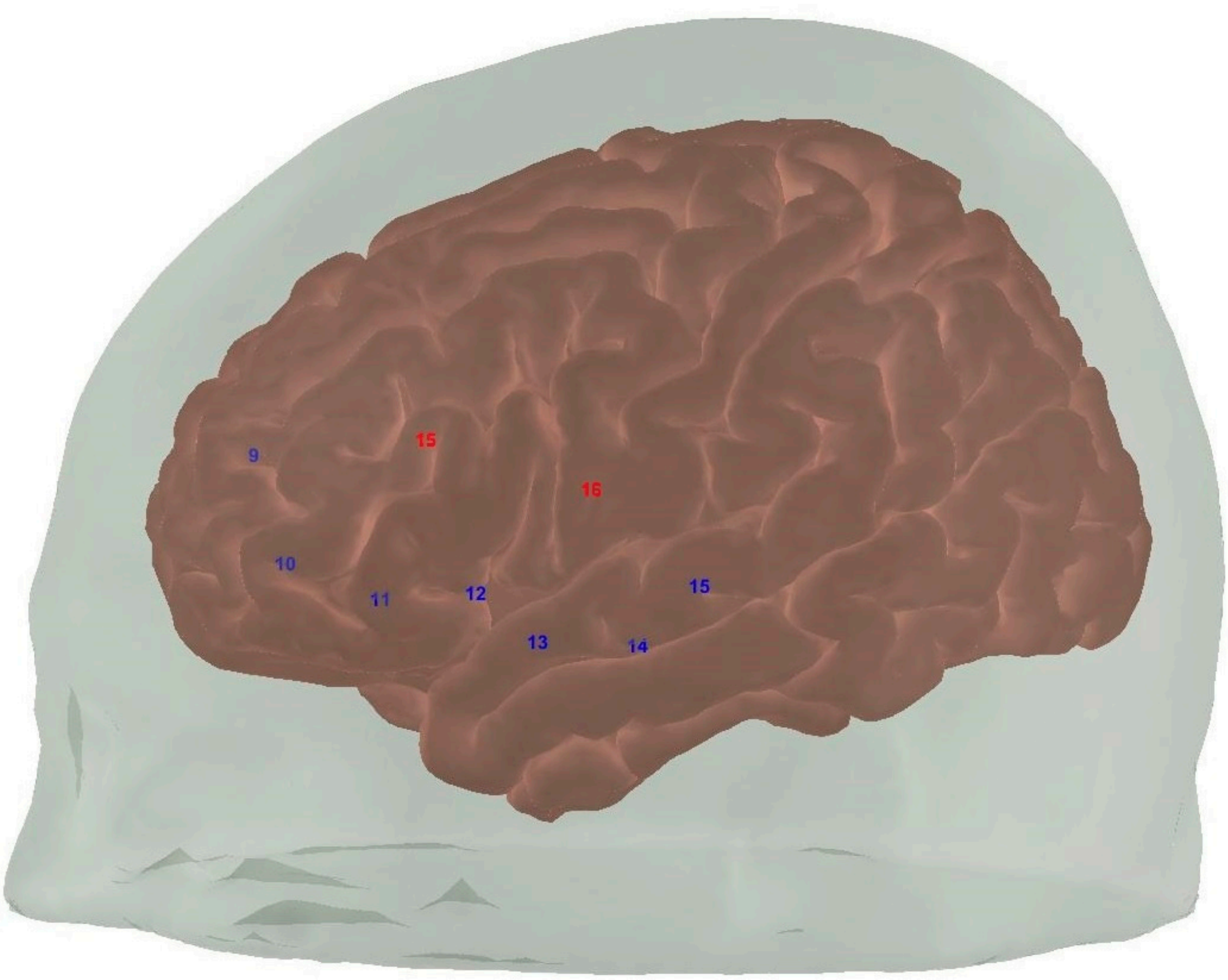

Note. Only the left hemisphere is shown. Blue numbers 9-15 correspond to the white channel numbers in Figure 3. Red numbers 15 and 16 correspond to the red dot sources in Figure 3. A Polhemus digitizer was used to collect the digitized points from experiment participants. The numbers presented in this figure are the median values of the digitized points collected at each optode location.

Figure S3. Tensor construction and decomposition

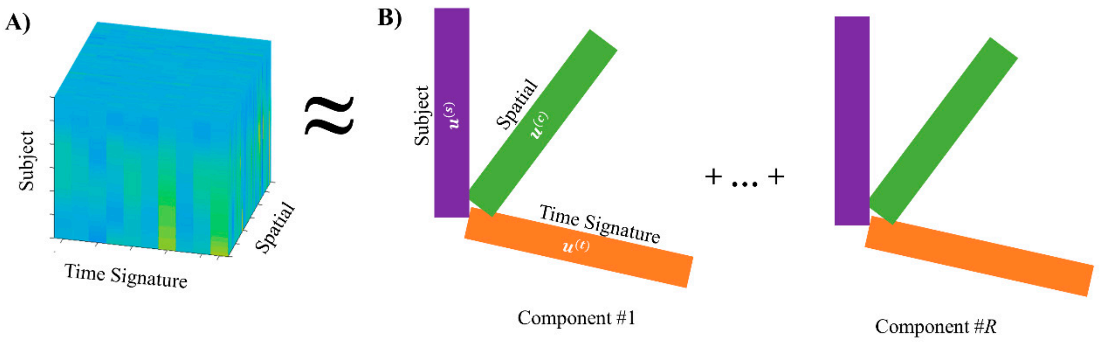

Note. (A) Illustrates the construction of a three-way tensor with time signature × spatial × subject modes. (B) Illustrates the implementation of canonical polyadic decomposition (CPD) on the tensor into number of time signature, spatial, and subject subcomponents from each mode.

Figure S4. Pipeline for the preprocessing of the fNIRS signal (in blue), tensor decomposition (in green), and validation of tensor decomposition with grand averaging (in orange).

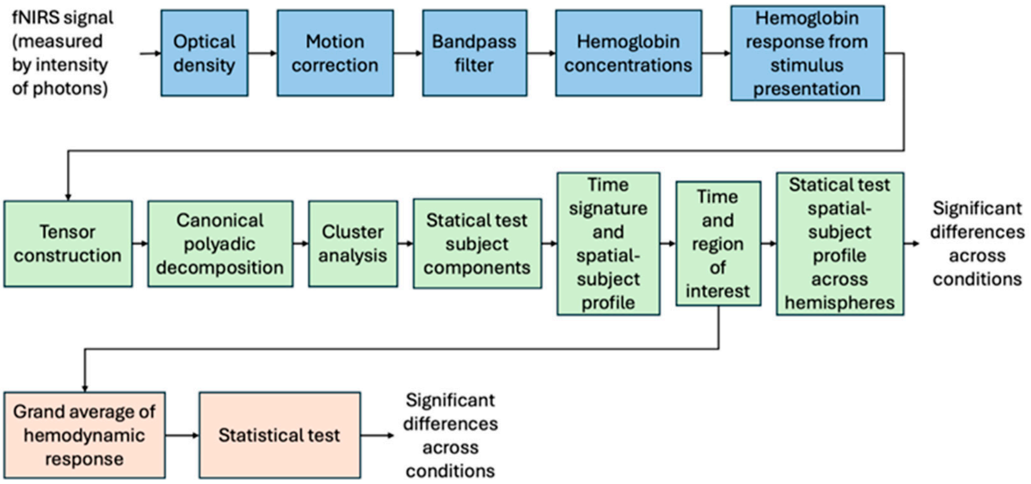

Figure S5. A magnified version of Figure 4, subfigure (C) – the spatial mode weights of left hemisphere component 1.

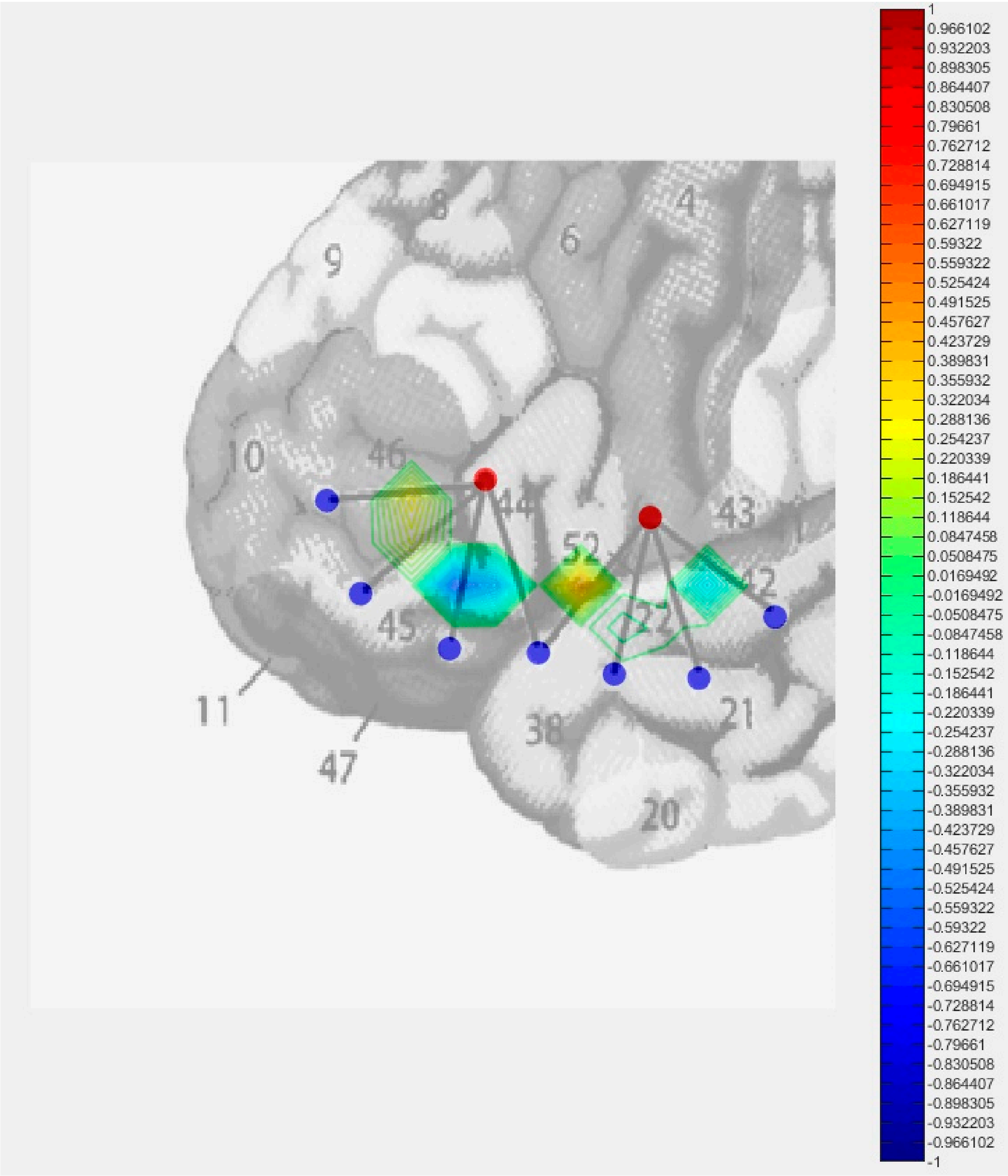

Supplement: Supplementary file 1 [file brainsci-15-00127-s001.zip › brainsci-3398735-supplementary.pdf]
